# Freeze-Dried Oat- and Rice-Based Porridges for Military and Emergency Rations: Formulation, Long-Term Storage Stability, Rheological Behaviour and Exploratory Sensory Evaluation

**DOI:** 10.3390/foods15213829

**Published:** 2026-10-26

**Authors:** Martin Szotkowski, Jiri Malisek, Kristina Trenzova, Katerina Kristofova, Monika Hartmannova, Blanka Dostalova

**Affiliations:** 1 Laboratory of Food Quality and Safety Research, Department of Logistics, Faculty of Military Leadership, University of Defence, Kounicova 65, 662 10 Brno, Czech Republic; jiri.malisek@unob.cz (J.M.); katerina.kristofova@unob.cz (K.K.); 2 Department of Plant Origin Food Sciences, Faculty of Veterinary Hygiene and Ecology, University of Veterinary Sciences, Palackeho tr. 1946/1, 612 42 Brno, Czech Republic; 3 Central Military Veterinary Institute, Ministry of Defence, Opavska 771/29, 748 01 Hlucin, Czech Republic; hartmannova.m@mo.gov.cz (M.H.); dostalova.b@mo.gov.cz (B.D.)

**Keywords:** freeze-dried porridge, oat porridge, rice porridge, military ration, emergency food, aluminium laminate packaging, shelf life, water activity, rheology, sensory evaluation

## Abstract

Lightweight, shelf-stable and acceptable meal components are required for military, emergency and humanitarian feeding systems. This study evaluated freeze-dried oat- and rice-based porridges, formulated as three recipe variants per matrix and packed in vacuum-sealed PET/ALU/PE laminate pouches. Samples were stored at 5, 25 and 40 °C for up to 18 months and assessed for physicochemical stability, rehydration-relevant rheology, microbiological safety, chemical composition and exploratory sensory quality. Freeze-drying reduced water activity to a very low range, and low aw, stable near-neutral pH and microbiological monitoring indicated no storage-related microbial outgrowth. Oat formulations contained higher protein and lipid fractions, whereas rice formulations were more carbohydrate-dominant, with no major macronutrient drift at the 12-month endpoint. Rheology revealed clear matrix-dependent behaviour: oat porridges were more viscous at comparable nominal formulation levels, whereas rice porridges showed stronger temperature-dependent thickening during heating. Exploratory condition-level analyses showed crude associations between apparent viscosity and positive sensory scores, particularly in rice porridges, but these associations were not independently distinguishable after adjustment for formulation, storage month and storage temperature. The results show that freeze-dried porridges may serve as ration components under the investigated conditions when formulation balances low-aw safety, structural stability, rehydration behaviour and exploratory sensory quality.

## 1. Introduction

Military and emergency food systems require meal components that are lightweight, compact, microbiologically safe, nutritionally meaningful and acceptable under stressful or logistically constrained conditions. Ration design is therefore not only a matter of nutrient delivery, but also of maintaining actual consumption, usability and sensory motivation during deployment. Long-term military ration research has shown that intake from packaged rations can decrease when foods are monotonous, poorly accepted or inconvenient, while acceptability, variety, serving temperature and preparation context strongly influence consumption [1]. Recent reviews similarly emphasize that operational rations should be assessed through nutritional, technological and sensory quality combined, particularly in extreme environments and field-feeding contexts [2,3].

Freeze-drying is well suited to high-value ration components because water is removed primarily by sublimation from the frozen state. Compared with conventional hot-air drying, freeze-drying generally results in lower thermal damage, better preservation of porous structure, improved rehydration and higher retention of colour and flavour attributes [4,5]. These advantages are especially relevant for composite cereal–fruit meals, where the consumer-relevant quality of the product is formed only after hot-water rehydration and is strongly influenced by texture, appearance, aroma and flavour.

The evidence base directly relevant to freeze-dried porridges is still fragmented. Freeze-dried rice porridge has been studied mainly with emphasis on freezing temperature, pore structure, rehydration and water-vapour adsorption [5], whereas oat porridge and oat bran systems have mostly been investigated through the physicochemical functionality of beta-glucan during freezing, freeze-drying and processing [6,7,8]. Direct long-term, factor-matched comparisons of freeze-dried oat and rice porridges under the same formulation, packaging, storage and rheological design remain limited. This gap is important because oat and rice matrices are likely to differ not only in nutritional profile, but also in rehydration behaviour, thermal response and sensory acceptability. The specific novelty of the present study is the direct comparison of oat- and rice-based freeze-dried porridges within one common long-term packaging, storage, rehydration and analytical framework.

The primary preservation principle of freeze-dried foods is the reduction of water activity rather than sterilization. Low aw limits microbial growth and slows many deterioration reactions; however, low-moisture foods may still carry viable microorganisms for long periods if contamination occurs before or after processing [9,10,11]. Consequently, shelf-life evaluation of freeze-dried ration components should combine aw, moisture content, packaging integrity and microbiological monitoring rather than relying on drying alone.

Packaging is a second critical determinant of shelf life. Freeze-dried products are highly hygroscopic because their porous structure and low equilibrium moisture content create a strong driving force for moisture uptake. Aluminium-containing multilayer laminates are therefore commonly used for long-term storage because they provide strong barriers to water vapour, oxygen, light and aroma compounds, helping to maintain low aw, reduce oxidative reactions and limit colour and flavour deterioration during storage [12,13]. In the present study, vacuum-sealed PET/ALU/PE laminate packaging was used to protect the porridges during cold, ambient and accelerated storage.

Breakfast-type porridges are attractive as military and emergency ration components because they can be prepared rapidly, consumed easily, rehydrated with hot water and used when appetite may be reduced. Oat and rice matrices also differ substantially in their structure-forming mechanisms. Oat products contain beta-glucans, insoluble fibre, proteins and lipids, and the viscosity of oat beta-glucan depends strongly on concentration, solubility and molecular weight [7,14,15]. These components can contribute to a stable, spoonable texture and may improve mouthfeel after rehydration.

Oat matrices may also be more sensitive to oxidative and hydrolytic quality changes because oats contain a relevant lipid fraction. Long-term storage studies on oat products have linked storage to rancidity-related sensory descriptors such as earthy, musty and rancid notes [16], and recent work on oat flour shows that hydrolytic and oxidative rancidification remain important, even when heat treatment is used to improve storability [17]. For oat-based ration meals, microbiological stability is therefore not the only shelf-life endpoint; lipid-related sensory drift must also be considered.

Rice-based porridges are dominated by starch behaviour. During heating in excess water, rice starch granules swell, lose crystalline order and gelatinize, while subsequent cooling and storage can promote molecular reorganization and retrogradation [18,19]. Rice porridge viscosity is strongly concentration-dependent, and the systems typically show shear-thinning behaviour [20]. In mixed starch–hydrocolloid systems, beta-glucans and oat flour can alter rice-starch pasting, retrogradation and rheology [21,22,23], supporting the expectation that oat and rice matrices will differ in their rehydrated texture and thermal response.

For ration development, rheological behaviour is particularly important because the product is consumed after rehydration, often at elevated temperature. Apparent viscosity at consumption-relevant temperatures can influence perceived thickness, spoonability, satiety and overall acceptability. Instrumental rheology also provides objective descriptors of structural changes caused by drying, storage, and reheating, complementing sensory texture evaluation [24]. In this context, viscosity retention during storage and the temperature response during heating are useful indicators of practical product robustness.

Colour and sensory quality are additional shelf-life endpoints. In fruit-containing cereal products, colour changes may reflect pigment degradation, non-enzymatic browning and moisture-related changes, and CIELAB-based colour difference is widely used to quantify visible changes during storage [25,26]. However, recent methodological work warns that universal perceptual thresholds for ΔE should be applied cautiously in food matrices unless the colour-difference formula and viewing context are specified [27]. Sensory analysis remains essential because instrumental stability does not automatically guarantee acceptability in the field; attributes such as taste, aroma, consistency and overall impression are directly linked to ration consumption [1,28].

The objective of this study was to formulate and evaluate freeze-dried oat- and rice-based porridges intended for military, emergency and humanitarian rations. The study directly compared oat- and rice-based matrices and the tested formulation variants within the same packaging and storage design, evaluating physicochemical stability, rheological behaviour after rehydration, microbiological safety, chemical composition and exploratory sensory quality. The working hypothesis was that oat and rice matrices would differ not only in absolute viscosity, but also in their thermal response, storage-induced structural evolution and exploratory sensory response, and that these differences would influence formulation selection for ration use.

## 2. Materials and Methods

### 2.1. Formulation of Oat and Rice Porridges

Six porridge formulations were prepared: three oat-based variants (Oat10, Oat15 and Oat20) and three rice-based variants (Rice15, Rice20 and Rice25) [Table 1 and Table 2]. The numerical code denotes the nominal percentage of the main cereal component in the formulation. Whole milk, sugar, salt and strawberries were used to provide nutritional density and sensory quality. The mixtures were cooked until a porridge-like consistency was obtained and were then homogenized to a smooth mass before freezing. Because cereal level changed along with milk, sugar and strawberry quantities, the variants are interpreted as complete formulations rather than as an isolated cereal-concentration factor.

### 2.2. Freeze-Drying and Packaging

Prepared porridges were processed in an AMARU freeze-dryer (Lyotrade Freeze Dryers, s.r.o., Klecany, Czech Republic). The freezing stage lasted 4 h, with the condenser/freezing drum maintained at approximately −40 to −42 °C and the recorded system pressure at 45–50 kPa. This was followed by a 44 h freeze-drying stage under maximum vacuum (approximately 20–40 Pa), while the condenser temperature remained at approximately −40 to −42 °C. During the final 20 h of drying, the sample trays were heated to 30 °C. After drying, samples were vacuum-sealed in aluminium multilayer laminate pouches with a PET/ALU/PE structure (12/9/90 µm). The laminate was selected because high-barrier aluminium-containing materials limit moisture and oxygen transfer, which is essential for the stability of hygroscopic low-moisture foods. Vacuum packing was performed under sanitary laboratory conditions.

### 2.3. Stability Testing

The stability of the freeze-dried porridge meals was evaluated under three different storage temperatures: 5 °C (cold storage), 25 °C (ambient conditions) and 40 °C (accelerated ageing/tropical exposure simulation). Samples were stored in controlled-temperature chambers for up to 18 months. The stability design included the initial 0-month point and subsequent storage intervals used for individual analytical sets; for rheological testing, the available intervals were 1, 3, 6, 9, 12, 15 and 18 months, with the 1-month point available mainly for the 40 °C accelerated condition. Where individual analytical datasets covered shorter or different intervals, this is specified in the respective results tables.

The samples were incubated in an ES 252 incubator (Nüve, Ankara, Turkey) or equivalent controlled-temperature storage chambers. Storage chambers were monitored for temperature stability. Relative humidity (RH) was not actively controlled, but it was recorded periodically during storage. The RH values ranged from approximately 40–50% at 5 °C, 50–65% at 25 °C and below 10% at 40 °C because of the dry-heat conditions in the accelerated storage chamber.

To ensure packaging integrity, the aluminium multilayer laminate pouches were regularly inspected for possible leakage, seal failure or mechanical damage. Samples were removed at predefined intervals for physicochemical, rheological, sensory and microbiological analyses. Because RH fluctuations can influence the moisture uptake and chemical stability of freeze-dried foods, the potential impact of environmental RH variation was considered during data interpretation, particularly for water activity, moisture content, colour development and sensory texture changes. A concise overview of the study design and analytical domains is provided in Supplementary Table S2, and Supplementary Table S3 summarizes the processed datasets retained by the authors and available from the corresponding author upon reasonable request.

Individually packaged pouches represented the independent storage experimental units for physicochemical and other applicable analyses. When three storage replicates were available, *n* = 3 represented three separately packaged pouches under the same formulation, storage time and temperature. Rheology was handled differently: for each formulation × storage-time × storage-temperature condition, material from two independently stored pouches was combined to form one composite sample. At least three rheological runs were then performed on this composite; these runs were treated as technical replicates and were averaged to one condition-level rheological value. Each formulation was prepared as one large production batch and then divided into the individual storage pouches; replication was therefore at the packaged storage-unit level rather than at the independent production-batch level.

### 2.4. Sensory Analysis

All sensory procedures were performed in accordance with the relevant ethical, military and food-testing requirements applicable to the University of Defence and with the general logic of controlled hedonic evaluation described in ISO 11136:2014 [28]. The study protocol was approved by the Ethics Committee of the University of Defence (protocol No. 02/2025; approved on 30 May 2025). All participants provided written informed consent before participation. The ethics approval and consent documentation are retained in the study records.

Six finished porridge formulations (three oat-based variants and three rice-based variants) were subjected to sensory evaluation after lyophilization, rehydration and storage. The first sensory evaluation was performed after one month of storage at 40 °C. A second evaluation was performed after three months of storage at 5 °C, 25 °C and 40 °C. Additional sensory results from the available storage design were processed according to the same evaluation scheme, where applicable.

The same eight assessors, consisting of students and staff of the University of Defence, constituted the longitudinal panel used for the final inferential sensory analysis throughout the storage study (*n* = 8). Participants were selected based on availability, familiarity with military ration products and predefined eligibility criteria. The final analysed panel size was limited by laboratory and logistical constraints, but it was considered suitable for controlled exploratory ration screening and for non-parametric evaluation of ordinal sensory data.

Additional students/assessors participated in selected sensory sessions, but they did not evaluate the complete sample series across the study. Their observations were therefore excluded from the repeated-measures inferential dataset. Consequently, all sensory statistics reported in this manuscript refer to the final analysed panel (*n* = 8) and should be interpreted as exploratory operational sensory screening rather than as population-level consumer acceptance testing.

Inclusion criteria included age between 18 and 50 years, good general health, absence of known food allergies or intolerances relevant to the porridge formulations, and no reported disorders affecting taste or smell. Participants were asked to refrain from smoking, drinking coffee or consuming strongly flavoured foods for at least two hours before evaluation. Exclusion criteria included recent respiratory illness, ongoing medication affecting sensory perception, known sensitivity to any ingredient used in the samples or failure to follow pre-test instructions.

Each freeze-dried porridge sample was rehydrated by adding an amount of water equivalent to the water removed during freeze-drying, thereby restoring the original water content. Water at approximately 95 °C was added in a porcelain bowl, and the sample was manually mixed and homogenized with a standard spoon for 30 s. Before sensory evaluation, the rehydrated samples were maintained in a combi oven at 70–72 °C and presented in coded containers within a maximum of 5 min after removal from the oven. The evaluation form included hedonic ratings and intensity ratings based on predefined scales. In accordance with the sensory principles used for military ration testing, assessors evaluated appearance, consistency/texture, taste, aroma, overall impression and selected descriptors such as intensity of foreign flavour, acidity, saltiness, bitterness, rancidity and sweetness, where relevant.

The hedonic scale ranged from 1 to 9, where 1 represented an extremely unpleasant perception and 9 represented an extremely pleasant perception. Higher scores, therefore, corresponded to better perceived sensory quality. Because sensory data are ordinal and may show inter-individual variability, medians were used as robust summary statistics for hedonic attributes. The evaluated attributes are described above; the processed sensory outputs are retained in the study records and are available from the corresponding author upon reasonable request.

### 2.5. Microbiological Analysis

Microbiological analyses were conducted throughout the stability testing period to verify the safety and hygienic quality of the freeze-dried products after preparation, packaging and storage. The final checked microbiological dataset retained individual replicate results for total viable count, yeasts, moulds/fungi, Staphylococcus, Enterobacteriaceae and aerobic sporulates. Positive quantified counts were transformed individually to log10(CFU/g). Values reported below the laboratory threshold were retained as censored observations (for example, <1.00 or <2.00 log10(CFU/g), depending on the reporting limit), without arbitrary numerical substitution. Mixed quantified/censored replicate sets were retained transparently and were not forced into mean ± SD summaries when censoring prevented a defensible numerical summary.

#### 2.5.1. Total Viable Count

The total viable count was determined according to ČSN EN ISO 4833-1 [29]. Samples were weighed, diluted and homogenized under sterile conditions. Appropriate decimal dilutions were prepared, and aliquots of the sample dilutions were spread on suitable non-selective agar media in Petri dishes. Plates were incubated at 30 °C ± 1 °C for 72 h. After incubation, plates containing countable colonies were selected, and the microbial load was calculated using the dilution factor, expressed as colony-forming units per gram of sample (CFU/g). Each sample was analysed in replicate, and results were expressed as mean values with standard deviation, where applicable.

#### 2.5.2. Enterobacteriaceae

Enterobacteriaceae were quantified according to ČSN ISO 21528-2 [30]. The sample preparation procedure was identical to that used for the total viable count. Suitable decimal dilutions were inoculated onto selective agar media and incubated under the conditions specified by the standard. Plates with countable colonies were used for calculation, and the results were expressed as CFU/g. The absence or low occurrence of Enterobacteriaceae was used as an indicator of the hygienic quality and post-processing safety of the freeze-dried porridges.

#### 2.5.3. Yeasts and Moulds

Yeasts and moulds were analysed according to ČSN ISO 21527-2 [31]. One gram of sample was diluted with peptone salt solution to obtain an initial 1:10 dilution and homogenized in a stomacher bag. Aliquots of the appropriate dilutions were spread on agar plates containing Dichloran 18% Glycerol Agar (DG18). Plates were incubated at 25 °C for 5 days and checked for colony growth. Results were calculated using the inoculated volume and dilution factor, expressed as CFU/g.

#### 2.5.4. Staphylococcus

Staphylococcus was monitored as a hygiene indicator and reported according to the reporting-threshold notation used in the final microbiological worksheet. Results were expressed as CFU/g or as below-threshold values, depending on the outcome of the laboratory analysis.

#### 2.5.5. Aerobic Sporulates

Aerobic sporulates were monitored as an additional indicator of resistant spore-forming microbial background in the cereal- and milk-containing dried products. Results were reported as CFU/g or as below-threshold values, according to the final microbiological worksheet, and were interpreted together with water activity and storage trends.

### 2.6. Chemical Composition Analysis

Chemical composition analysis was performed to determine the total protein, fat and saccharide contents of the lyophilized porridge formulations. The analyses were performed in accordance with Czech and EU regulations for food products and food information, particularly Regulation (EU) No 1169/2011, and with regard to the nutritional evaluation requirements relevant to military ration development, including STANAG 2937 [32]. The chemical composition data were used to assess the nutritional adequacy of the products as components of military or emergency food rations.

#### 2.6.1. Total Protein Content

Total protein content was determined using the Kjeldahl method according to an internal laboratory SOP based on ČSN ISO 5983-1 [33]. A homogenized sample was digested with concentrated sulphuric acid in the presence of a catalyst, converting nitrogen-containing compounds into ammonium sulphate. After digestion, the solution was alkalized with sodium hydroxide, and the released ammonia was distilled and captured in a known volume of acid. The amount of nitrogen was quantified by titration. Protein content was calculated by multiplying the nitrogen content by the appropriate conversion factor and was expressed as a percentage of sample mass.

#### 2.6.2. Fat Content

Fat content was determined gravimetrically by Soxhlet extraction according to an internal laboratory SOP adapted from ČSN ISO 1443:1973 [34]. Approximately 10 g of homogenized sample was weighed, ground to a fine powder and placed in a cellulose extraction thimble. The sample was extracted for 90 min using 300 mL of extraction solvent. The extract was cooled, filtered and evaporated under vacuum, and the dried extract was weighed. Fat content was calculated gravimetrically and expressed as a percentage of sample mass.

#### 2.6.3. Saccharide Content

Total saccharide content was determined using an internal laboratory acid-hydrolysis and colorimetric procedure. Approximately 1 g of homogenized lyophilized sample was hydrolysed with acid solution at 90 °C for 2 h to convert complex carbohydrates into simpler sugars. After cooling, the hydrolysate was neutralized with sodium hydroxide and filtered. Total saccharides were quantified using the phenol-sulphuric acid method, as described by Dubois et al. [35]. Absorbance was measured spectrophotometrically at 490 nm, and concentrations were calculated using a glucose calibration curve. Results were expressed as a percentage of the original sample mass.

Chemical composition values were expressed as percentages of the lyophilized product. In the current analytical dataset, complete compositional values were available for the initial products and for the 12-month endpoint at the storage temperatures of 5, 25 and 40 °C. These data were therefore interpreted primarily as formulation verification and endpoint compositional stability information rather than as a full kinetic description across the entire storage period.

### 2.7. Physical Properties Analysis

The physical stability of the freeze-dried porridge samples was evaluated by measuring pH, water activity, moisture content and colour parameters. These characteristics are essential for understanding product stability because they influence microbial growth, chemical reactions, texture development, colour changes and overall sensory quality during storage. The following subsections describe the methods used for each physical parameter.

#### 2.7.1. pH Analysis

pH was determined potentiometrically using an internal laboratory SOP for aqueous suspensions of lyophilized cereal products. One gram of lyophilized sample was weighed and dispersed in 100 mL of distilled water to obtain a homogeneous suspension. The mixture was thoroughly stirred to ensure hydration and dispersion of the sample. pH was measured using a calibrated pH meter HI 99165 (Hanna Instruments Inc., Woonsocket, RI, USA) equipped with a glass electrode. Calibration was performed before measurement using standard buffer solutions at pH 4.00 and 7.00. Measurements were taken after stabilization of the reading. For each storage condition, three independently stored pouches were analysed, with one pH determination obtained from each pouch; the three pouch-level values were summarized as mean ± SD.

#### 2.7.2. Water Activity Analysis

Water activity (aw) was measured according to ISO 18787:2017 [36]. Approximately 1 g of ground lyophilized sample was placed into a suitable sample cup and allowed to equilibrate at room temperature before analysis. Measurements were performed using a Waterlab aw analyser (Steroglass S.r.l., Perugia, Italy), calibrated with standard solutions of known water activity. After insertion of the sample cup, the instrument was allowed to stabilize before the value was recorded. For each storage condition, water activity was measured once in each of three independently stored pouches; the three pouch-level values were summarized as mean ± SD. Water activity values were used to evaluate the susceptibility of the product to microbial growth and moisture-related quality changes. Values below approximately 0.6 are generally considered strongly restrictive for the growth of most microorganisms in low-moisture foods.

#### 2.7.3. Moisture Content

Moisture content was determined gravimetrically according to an internal laboratory SOP adapted from ISO 1442:1997 [37]. Approximately 5 g of homogenized sample was placed in a previously dried and weighed container. The samples were dried in an SF55 hot-air dryer (Memmert, Schwabach, Germany) at 103 ± 2 °C for 6 h or until constant mass was achieved. After drying, containers were cooled in a desiccator and weighed. Moisture content was calculated from the difference in mass before and after drying. For each storage condition, moisture content was determined independently in three stored pouches, with one gravimetric determination per pouch; the three pouch-level values were summarized as mean ± SD. Moisture was calculated as moisture (%) = [1 − (post-drying mass − tare mass)/sample mass] × 100. During the data audit, previously negative spreadsheet-derived values were traced to the original gravimetric records and recalculated directly from the recorded tare mass, sample mass and post-drying mass. Summary standard deviations were recalculated consistently as sample SD (n − 1); the original raw records and all corrections were retained in the QC log for traceability.

Moisture content is a key indicator for freeze-dried foods because these products are hygroscopic and can absorb water from the surrounding environment if packaging integrity or barrier properties are insufficient. Moisture content was therefore evaluated together with water activity to assess storage stability, texture preservation and microbiological safety.

#### 2.7.4. Colour Measurement

Colour was measured using a ColorFlex EZ spectrophotometer (HunterLab, Reston, VA, USA). The instrument was calibrated using white and black calibration tiles before measurement. A green calibration tile (X = 15.32, Y = 21.46, Z = 16.25) was used as a control standard during the measurement series. Measurements were performed at room temperature. Samples were ground to ensure homogeneity, and for each analysed pouch portion, at least four instrumental colour readings were obtained; these readings were treated as technical replicates and averaged at the pouch level before condition-level summaries.

Colour was expressed in the CIELAB colour space using L* (lightness), a* (red-green chromatic coordinate) and b* (yellow-blue chromatic coordinate). Total colour difference (ΔE) was calculated relative to the appropriate fresh or reference sample. Larger ΔE values indicate greater colour change. Because universal sensory-perceptibility thresholds for ΔE are not validated for this specific food matrix and viewing context, ΔE was interpreted as an objective marker of colour drift rather than as a direct sensory-acceptance threshold [27].

### 2.8. Rheological Analysis

Rehydrated porridge samples were measured using a rotational rheometer equipped with a coaxial-cylinder geometry. The main measurement used for comparison was a temperature-ramp test performed at a constant shear rate of approximately 77.3 s^−1^. Apparent viscosity was extracted at approximately 25, 40 and 65 °C from the main ramp interval. The 25 °C point was consistently extracted from the main ramp section rather than from the initial stabilization phase. The selected shear rate was intended to approximate the shear generated during practical spoon mixing/stirring of the rehydrated porridge and to provide a standardized, consumption-relevant comparative condition during the temperature ramp; it was not intended as an exact surrogate for in-mouth shear.

The 40 °C viscosity was used as a practical indicator of consistency during mild heating, whereas the 65 °C viscosity represented behaviour at a higher consumption-relevant temperature. For each sample, the thermal viscosity response ratio (TVR) was calculated as η65/η25 × 100. A value below 100% indicates that apparent viscosity at 65 °C was lower than at 25 °C, whereas a value above 100% indicates a net increase in apparent viscosity across the defined temperature interval. TVR is an author-defined descriptive ratio and should not be interpreted as a direct measure of thermal stability; possible structural mechanisms are considered only as interpretations. Viscosity retention indices (VRI) were calculated relative to the corresponding 0-month baseline: VRI = ηt/η0M × 100. Values below 100% indicate a decrease relative to baseline, whereas values above 100% indicate increased viscosity or structural reorganization during storage.

### 2.9. Statistical Analysis

Data were summarized as mean ± standard deviation where replicate measurements were available. Physicochemical measurements were summarized according to their independent-pouch replication. For rheology, the ≥3 technical runs obtained from each two-pouch composite were first averaged to one formulation × storage-month × storage-temperature condition-level value before between-condition, sensory–rheology and PCA analyses. Matrix effects between oat and rice porridges were evaluated at common nominal formulation levels (15 and 20) using Welch tests for the condition-level composite means because variances were not assumed to be equal. Because other formulation components changed concurrently, these comparisons are interpreted as contrasts between complete formulations rather than as isolated cereal-concentration effects. Effect size was expressed as Hedges’ g. Because VRI and TVR are not standardized cereal-porridge descriptors in the literature, all index values were interpreted as author-defined descriptive indicators and were reported together with the underlying apparent viscosity values. Technical rheological runs were not treated as independent observations in any between-condition inferential analysis.

Sensory data were treated as ordinal repeated measures. Within each matrix × storage-month × storage-temperature × sensory-attribute session, the three formulations were compared using Friedman’s test, with assessor as the blocking factor [38]. Significant omnibus tests were followed by paired Wilcoxon signed-rank tests for the three formulation contrasts [39], with Holm adjustment [40]. Kendall’s W was reported as the omnibus effect size. At incomplete 9-month sessions, only assessors with complete scores across all three formulations were included, and the effective paired n was retained. A Benjamini–Hochberg false-discovery-rate analysis across the larger family of sensory tests was used as a sensitivity analysis.

Formulation-level differences were evaluated descriptively from pooled matched data and from the session-specific repeated-measures analysis. Because cereal level, milk, sugar and other recipe components changed simultaneously, formulation was treated as a categorical factor, and no causal effect was attributed to cereal concentration alone. Changes in the magnitude of formulation differences across storage time and temperature were interpreted descriptively.

Associations between sensory attributes and instrumental rheological descriptors were first summarized using condition-level Spearman rank correlations as exploratory unadjusted associations. To reduce confounding by formulation and storage condition, rheological replicates were aggregated to formulation × month × storage-temperature condition cells. The primary reviewer-driven analysis used the balanced 3–18 month window at 5, 25 and 40 °C (54 condition cells per matrix). Separate matrix-specific models related condition-level sensory score to ln(η40), formulation, storage month and storage temperature; formulation, month and temperature were categorical factors, and HC3 robust standard errors were used. η65, TVR and VRI were evaluated only as secondary descriptors. Sensitivity analyses repeated the primary models after exclusion of the incomplete 9-month sensory sessions.

Principal component analysis (PCA) was repeated using the final QC-checked condition-level dataset. The primary PCA window comprised 3–18 months at 5, 25 and 40 °C (108 condition-level observations). Apparent viscosity at 40 °C was natural-log transformed, and all active variables were standardized to z scores using the sample standard deviation. To limit redundant weighting of highly correlated rheological measures, ln(η40), TVR and VRI η40 were retained. PCA1 combined water activity, pH, moisture, ΔE of the lyophilized product and the three rheological descriptors. PCA2 combined pH, rehydrated ΔE, the three rheological descriptors and condition-level mean sensory scores for appearance, consistency, taste/aroma and overall impression. Rehydrated ΔE rows omitted during an earlier merge were restored from the validated colour source after cross-checking against 97 overlapping values (maximum absolute difference 0.0005 ΔE, attributable to rounding); no imputation was used. A sensitivity analysis excluding 9-month observations was performed. PCA was interpreted as an exploratory description of covariance structure and clustering rather than as evidence of causal relationships or formulation optimization.

For the integrated sensory–rheological analysis, each row represented one formulation × storage month × storage-temperature condition after aggregation of technical rheological runs from the two-pouch composite to one condition-level value and calculation of the corresponding condition-level sensory mean. The balanced primary dataset comprised 54 condition cells per matrix (108 in total) for 3, 6, 9, 12, 15 and 18 months at 5, 25 and 40 °C. This condition-level structure avoids treating technical readings or multiple measurements from the same storage condition as additional independent observations.

## 3. Results

### 3.1. Overall Stability Profile and Analytical Scope

The study generated an integrated stability dataset covering freeze-dried oat and rice porridges prepared as three formulation variants per matrix and stored at 5, 25 and 40 °C. The results are interpreted as a combined shelf-life dataset rather than as isolated analytical endpoints because the long-term suitability of dried ration components depends on the simultaneous control of water activity, moisture content, colour, microbiological safety, rehydration behaviour and exploratory sensory quality [1,12]. Rheological measurements were particularly informative because they described the practical texture of the rehydrated porridges during heating, which is close to the intended preparation and consumption scenario.

Across the integrated dataset, oat and rice formulations differed most clearly in rehydrated rheology, while water activity remained low, and microbiological monitoring did not show progressive storage-related outgrowth. Mechanistic interpretation of the oat and rice matrix differences is addressed in Section 4.2 and Section 4.3.

The direct same-design comparison of the two cereal matrices is interpreted together with the relevant mechanistic literature in Discussion Section 4.2 and Section 4.3.

### 3.2. Physicochemical Stability: Water Activity, Moisture, pH and Colour

Fresh cooked porridges exhibited high water activity, as expected for ready-to-eat hydrated cereal systems(Table 3). After freeze-drying, aw decreased sharply and generally remained within the low range typical of highly stable dried foods. Most lyophilized values were approximately 0.07–0.13, far below aw = 0.60, which is commonly treated as a practical boundary below which microbial growth is strongly restricted in low-moisture foods [9,11,12]. The drying process therefore achieved the main preservation target of converting a high-moisture cooked porridge into a low-aw ration component. Nevertheless, low aw should be interpreted as growth-inhibiting rather than sterilizing, because microorganisms may survive in low-moisture foods even when growth is prevented [10,11].

The pH values remained close to neutrality throughout storage. Oat porridges ranged from approximately 5.73 to 6.06 and rice porridges from 5.72 to 6.07. Such pH values are not, by themselves, strongly inhibitory to microorganisms; therefore, the microbiological safety of the products is primarily attributable to low aw, hygienic processing and high-barrier packaging rather than to acidity. The absence of marked acidification or alkalinization also indicates that no major fermentation-like or alkaline degradation processes were evident in the physicochemical dataset.

Colour change was more informative than pH for describing product evolution during storage. Total colour difference (ΔE*ab) increased mainly under storage stress, especially in fruit-containing formulations. The largest ΔE*ab changes were observed under storage stress. Possible pigment-, browning- and moisture-related mechanisms are considered in Section 4.6 rather than inferred directly from the colour measurements. However, ΔE*ab should be presented as an objective colour-drift marker rather than a direct sensory rejection threshold unless the formula and perception context are explicitly specified [27].

**Figure 1:**
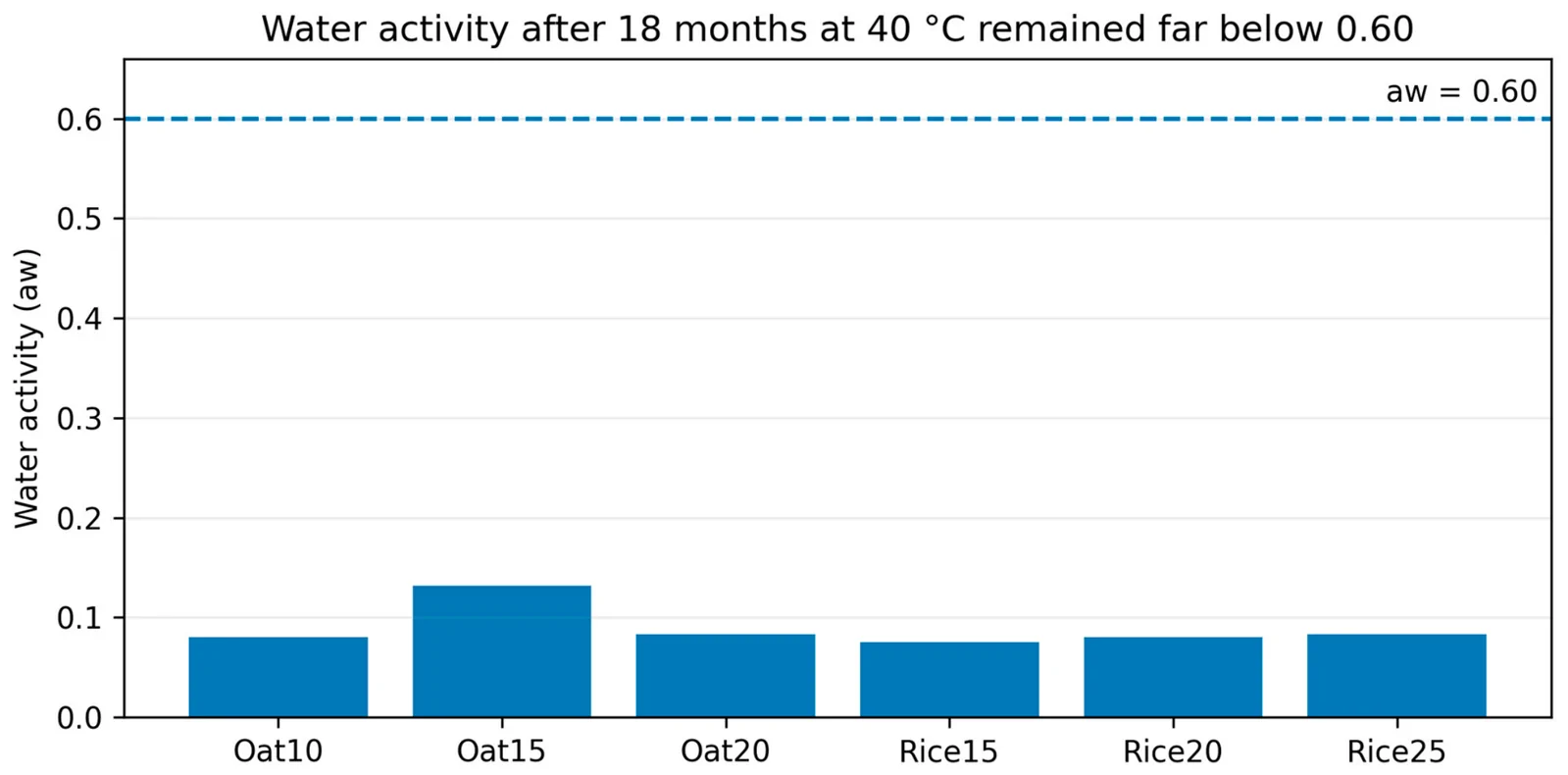
Water activity of selected lyophilized porridge formulations after 18 months of storage at 40 °C. The dashed reference line indicates aw = 0.60, which is commonly used as a practical threshold below which growth of most microorganisms is strongly restricted in low-moisture foods.

The 18-month endpoint at 40 °C provides a useful stress-condition illustration of the physicochemical stability of the products (Figure 1) and their packaging solution. All shown formulations remained far below aw = 0.60, supporting the microbiological stability observed during storage.

**Figure 2:**
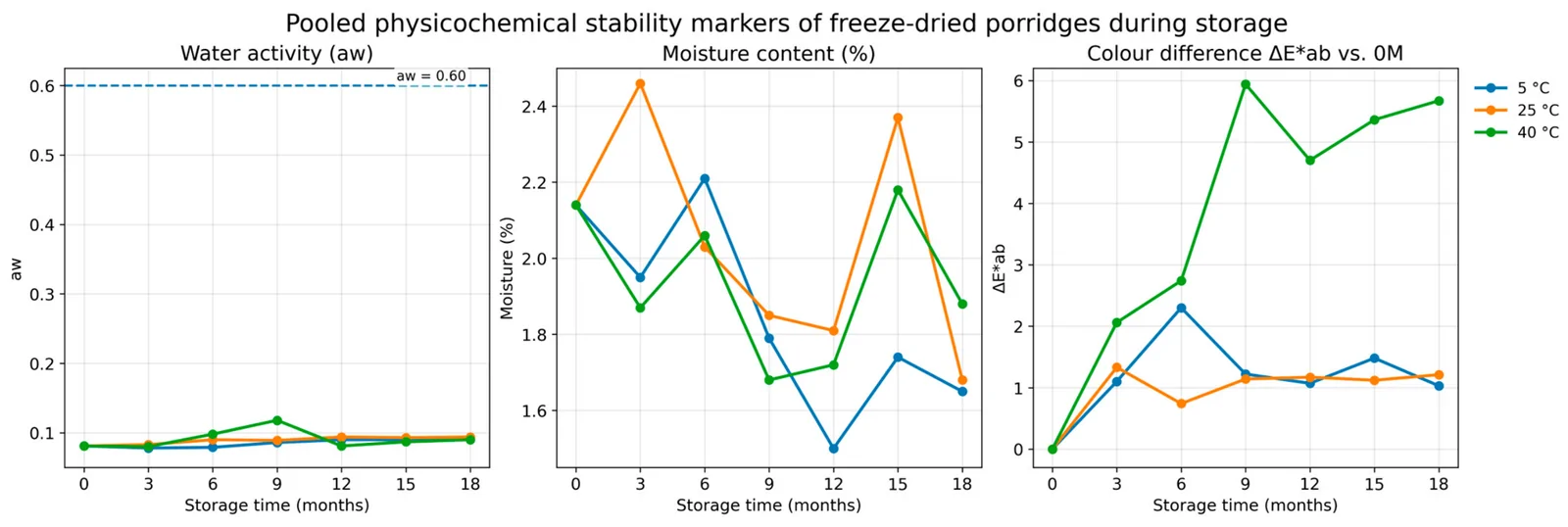
Pooled physicochemical stability markers of freeze-dried porridges during storage. The panels show water activity, moisture content and colour difference (ΔE*ab versus 0M) as pooled means across all formulations. The dashed line in the water-activity panel indicates aw = 0.60. The moisture panel uses the gravimetrically recalculated moisture dataset described in Section 2.7.3.

The broader physicochemical development of the freeze-dried products is summarized in Figure 2. Across the monitored period, pooled water activity remained far below aw = 0.60, while moisture values stayed low after recalculation of the previously erroneous spreadsheet-derived moisture values obtained from the original gravimetric records. Colour difference increased mainly under storage stress, supporting the interpretation that visible and structural changes, rather than microbial growth, were the most informative shelf-life endpoints. The extended supporting trend visualization is provided in Supplementary Figure S1, and the corresponding processed long-format datasets are summarized in Supplementary Table S3 and retained by the authors for access upon reasonable request.

### 3.3. Rheological Behaviour of Oat Porridges

The oat formulations showed a clear formulation-dependent increase in apparent viscosity. Oat20 was consistently the most viscous oat formulation, followed by Oat15 and Oat10. Oat20 had the highest apparent viscosity, followed by Oat15 and Oat10; possible compositional mechanisms underlying this pattern are considered in Section 4.3.

The 18-month data show that Oat10 remained the least viscous formulation. Its η40 values were clearly below those of Oat15 and Oat20, and under some conditions, it showed a non-classical thermal response. This pattern varied with formulation and storage condition; possible structural explanations are considered in Section 4.3. Oat15 represented an intermediate formulation, whereas Oat20 maintained the strongest practical viscosity profile after long-term storage.

Oat porridges generally exhibited thermal thinning during the ramp, particularly Oat15 and Oat20, as shown by TVR values below 100%. TVR values below 100% indicate only that apparent viscosity at 65 °C was lower than at 25 °C under the defined temperature ramp. Potential implications for hot consumption are discussed separately and require interpretation together with the absolute viscosity values.

### 3.4. Rheological Behaviour of Rice Porridges

Rice porridges had a lower absolute viscosity at moderate temperatures than that of oat porridges, especially at the common 15% and 20% formulation levels. The lower η40 values indicate that rice-based systems produced a lighter texture under mild heating. The observed η40 differences are reported here as matrix-level rheological findings; starch-based mechanisms are considered in Section 4.3.

Unlike the oat formulations, several rice porridges had η65 values higher than η40 and, in some cases, TVR values above 100% [Table 4]. This indicates a net increase in apparent viscosity between approximately 25 and 65 °C under the defined ramp; possible structural mechanisms are considered in Section 4.3. Implications for field preparation require direct validation under controlled water-dose, standing-time and serving-temperature conditions.

### 3.5. Direct Comparison Between Oat and Rice Matrices

For direct matrix comparison, only the common nominal formulation levels present in both matrices (15% and 20%) were used. During the 3–18 month storage period, oat porridges had substantially higher apparent viscosities than those of rice porridges. The mean η40 value was 11,902 mPa·s for oat matrices and 2899 mPa·s for rice matrices, corresponding to an approximately four-fold difference. The η65 values were also higher for oat porridges (8581 mPa·s) than for rice porridges (4024 mPa·s). These results show that oat and rice porridges cannot be treated as interchangeable cereal bases when rehydrated texture is a key ration-design endpoint.

The difference in absolute viscosity was highly significant (Welch tests, *p* < 0.001) and associated with large effect sizes. At the same time, rice porridges showed higher mean TVR and higher VRI η40 values than those of oat porridges. The higher VRI response should not be interpreted as evidence of better stability. It indicates a larger relative change from the formulation-specific baseline; mechanistic interpretation is considered in Section 4.4. The matrix-level Welch tests and Hedges’ g values reported in Table 5 were recalculated from 36 condition-level composite means per matrix (2 common nominal formulation levels × 6 storage months × 3 storage temperatures), after averaging technical runs within each composite.

**Figure 3:**
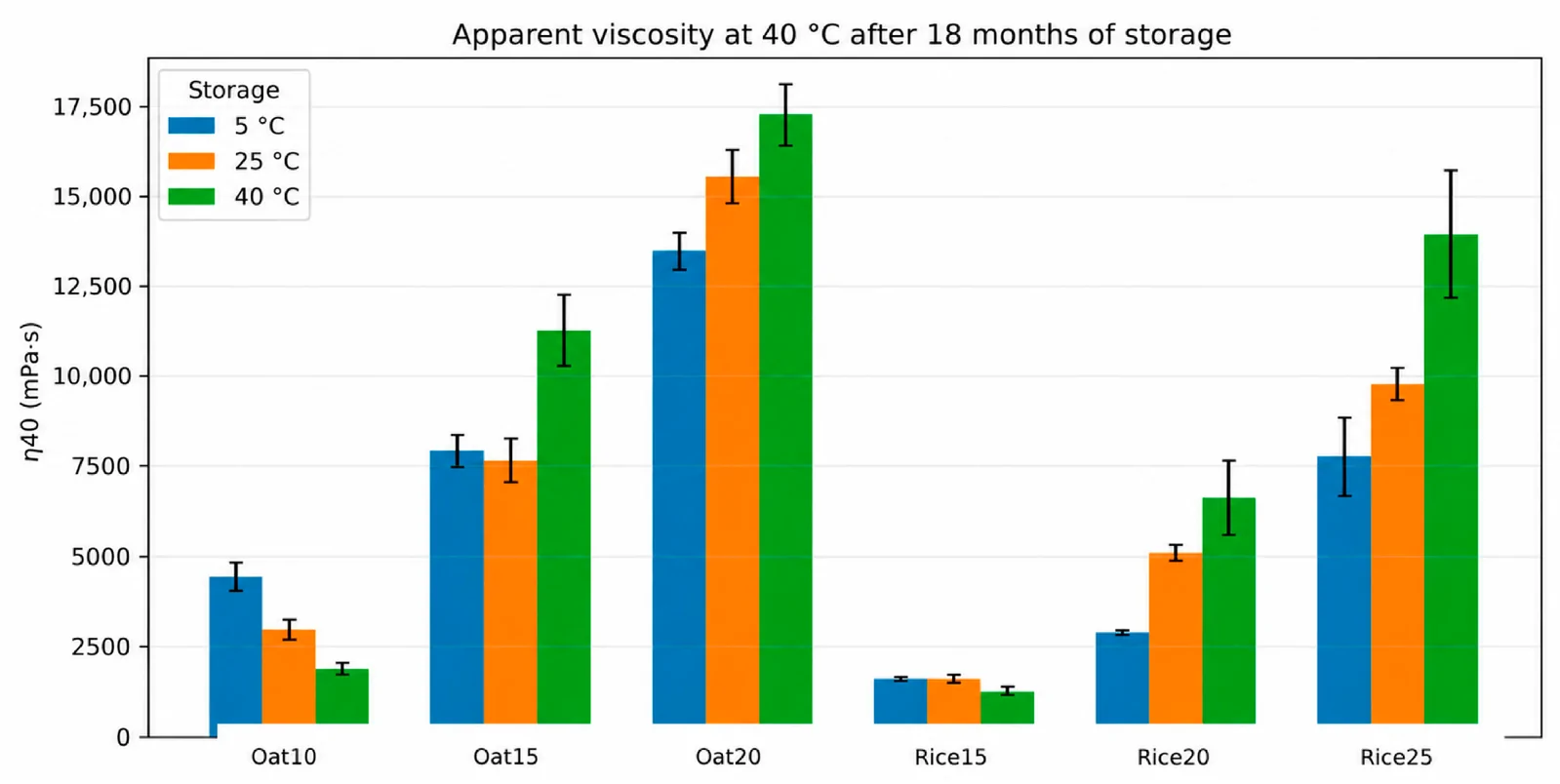
Apparent viscosity at 40 °C (η40) after 18 months of storage for oat and rice porridge formulations. Error bars represent standard deviations. The η40 parameter was used as a practical indicator of consistency at mild heating conditions relevant to rehydrated consumption. The error bars quantify variability among technical rheological runs of the two-pouch composite and should not be interpreted as variability among independent storage replicates.

The endpoint comparison after 18 months is shown in Figure 3. It clearly confirms the formulation-dependent increase in η40 and the matrix-dependent difference between oat and rice porridges, with Oat20 and Rice25 showing the highest viscosity within their respective matrices. A complementary endpoint visualization including both η40 and η65 at 18 months and 40 °C is provided in Supplementary Figure S2.

**Figure 4:**
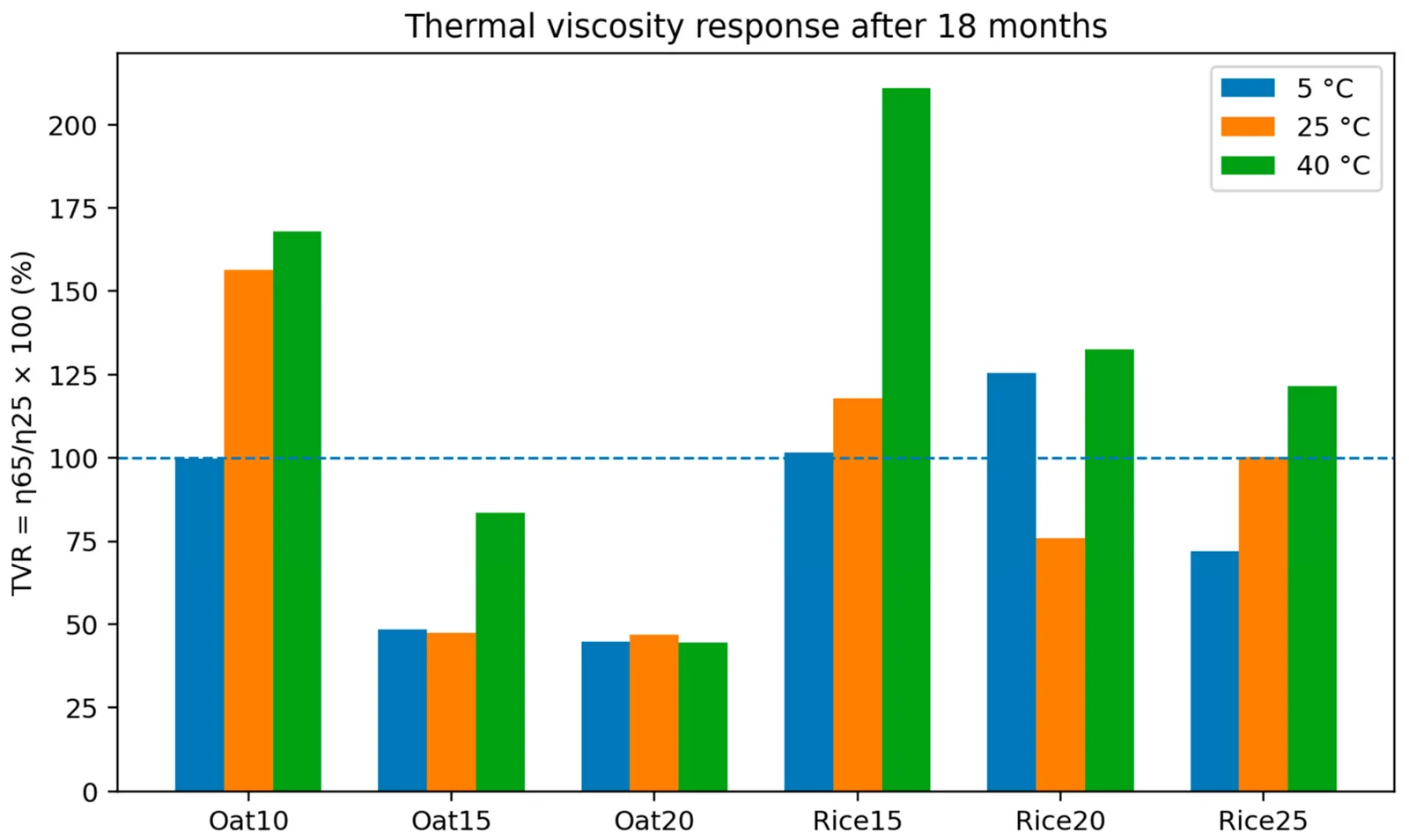
Thermal viscosity response ratio (TVR = η65/η25 × 100) after 18 months of storage. The dashed line at 100% separates thermal thinning (TVR < 100%) from a net increase in apparent viscosity across the defined temperature interval (TVR > 100%). Error bars represent standard deviations. These error bars likewise represent technical-run variability of the two-pouch composite.

The different thermal responses of the matrices are illustrated in Figure 4. Oat formulations generally remained below 100%, whereas several rice formulations exceeded 100%, especially under accelerated storage, showing a greater net increase in viscosity across the defined ramp for several rice formulations.

**Figure 5:**
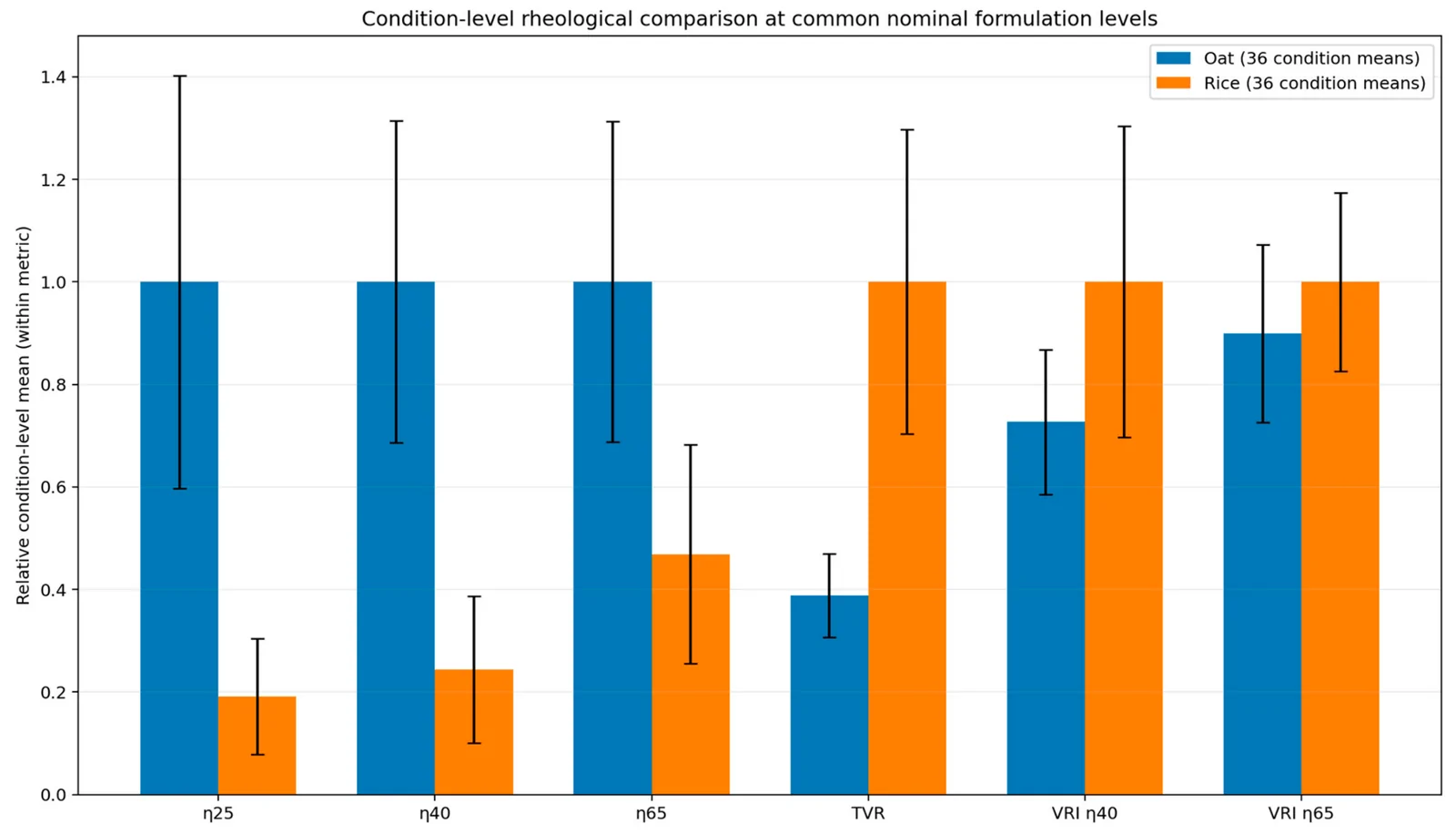
Condition-level comparison of oat and rice matrices at common nominal formulation levels (15 and 20) during the 3–18 month storage period. For each rheological metric, the two matrix means were scaled to the larger matrix mean to permit visualization on a common axis. Bars represent the mean of 36 condition-level composite values per matrix, and error bars represent the SD across these condition-level values. Each condition-level value is the mean of ≥3 technical runs from a composite formed from two stored pouches. Inferential statistics are reported in Table 5.

The pooled matrix-level statistical comparison is visualized in Figure 5. Oat matrices had markedly higher absolute viscosity values, whereas rice matrices showed higher TVR and VRI η40 values, reflecting differences in the thermal-response and storage-retention descriptors rather than a direct measure of stability. In the revised analysis, this visualization is based on condition-level composite means rather than pooled individual rheological runs.

### 3.6. Storage Temperature Effects and Long-Term Structural Evolution

Storage temperature influenced rehydrated viscosity, but the direction of this effect differed between formulations and matrices. The 40 °C condition acted as an accelerated ageing stress and revealed structural changes that were less apparent at 5 °C and 25 °C. Potential mechanisms underlying the storage-temperature effects are considered in Section 4.9.

For oat porridges, Oat20 showed the highest absolute viscosity after 18 months, while Oat15 showed intermediate values and Oat10 the lowest levels. For rice porridges, Rice25 had the highest absolute viscosity, whereas Rice15 and Rice20 showed larger TVR changes under some storage conditions. These observations describe formulation-specific rheological responses and do not identify a superior formulation.

### 3.7. Sensory Analysis Results

The final inferential sensory analysis was restricted to the same eight longitudinal assessors and treated the three formulations within each session as repeated measures. Across 560 session-specific Friedman tests, 224 (40.0%) had raw *p* < 0.05. Significant omnibus tests were followed by paired Wilcoxon signed-rank comparisons with Holm adjustment; 154 pairwise formulation contrasts remained significant. A conservative Benjamini–Hochberg false-discovery-rate sensitivity analysis retained 116 of the 560 omnibus tests. The most robust signals were concentrated in appearance, consistency, taste/aroma and overall impression. Because the panel was small and operational, these findings are interpreted as exploratory evidence of formulation differences within the tested panel rather than as population-level preference.

Descriptively, corrected pooled positive means followed Oat10 > Oat15 > Oat20 for the main positive attributes in the oat series, while Rice15 > Rice20 > Rice25 was observed in the rice series. These pooled values are used only to show the direction of the sensory profiles; inferential conclusions are based on the session-specific repeated-measures tests. Under the broader FDR sensitivity analysis, the main positive attributes remained the most robust signals, whereas several isolated effects in the secondary intensity descriptors were less stable.

Because the final analysed panel was small and the test was designed as an operational/exploratory ration assessment, the sensory results should be interpreted as within-panel evidence of formulation differences rather than as a definitive consumer preference study. The repeated-measures Friedman/Wilcoxon–Holm approach accounts for the dependence created by the same assessors evaluating all three formulations within a session [Table 6]. Future validation with a larger trained or semi-trained panel would strengthen the sensory conclusions [24].

**Figure 6:**
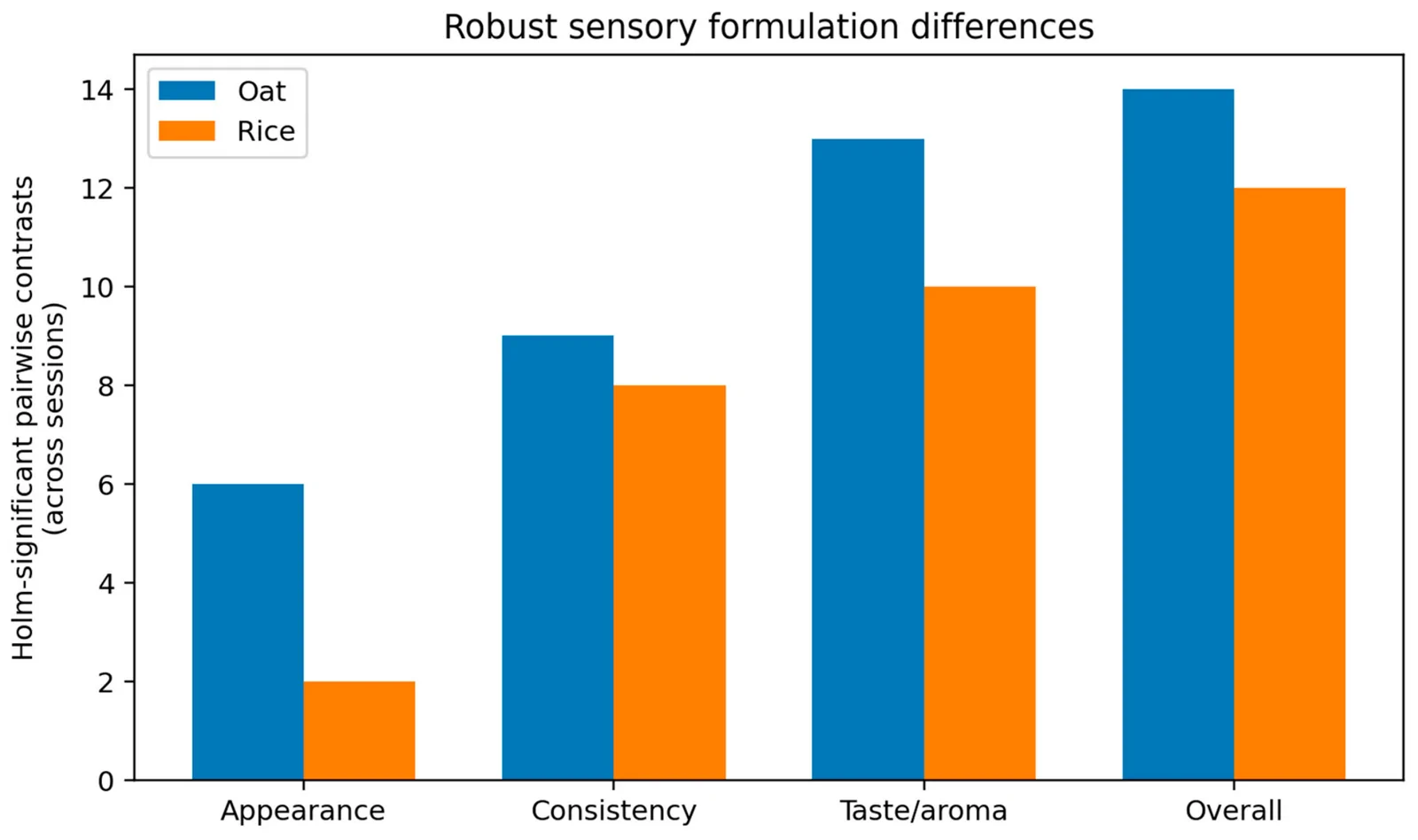
Sensory attributes most frequently associated with significant formulation differences. Bars show the number of Holm-significant paired Wilcoxon formulation contrasts obtained after significant Friedman tests for the main positive sensory block (appearance, consistency, taste/aroma and overall impression). The counts are descriptive summaries of the repeated-measures analysis and do not represent independent consumer-preference estimates.

The distribution of Holm-significant pairwise formulation contrasts is summarized in Figure 6. The largest counts occurred for the main positive sensory attributes; the figure is a descriptive summary of the repeated-measures post hoc results.

**Figure 7:**
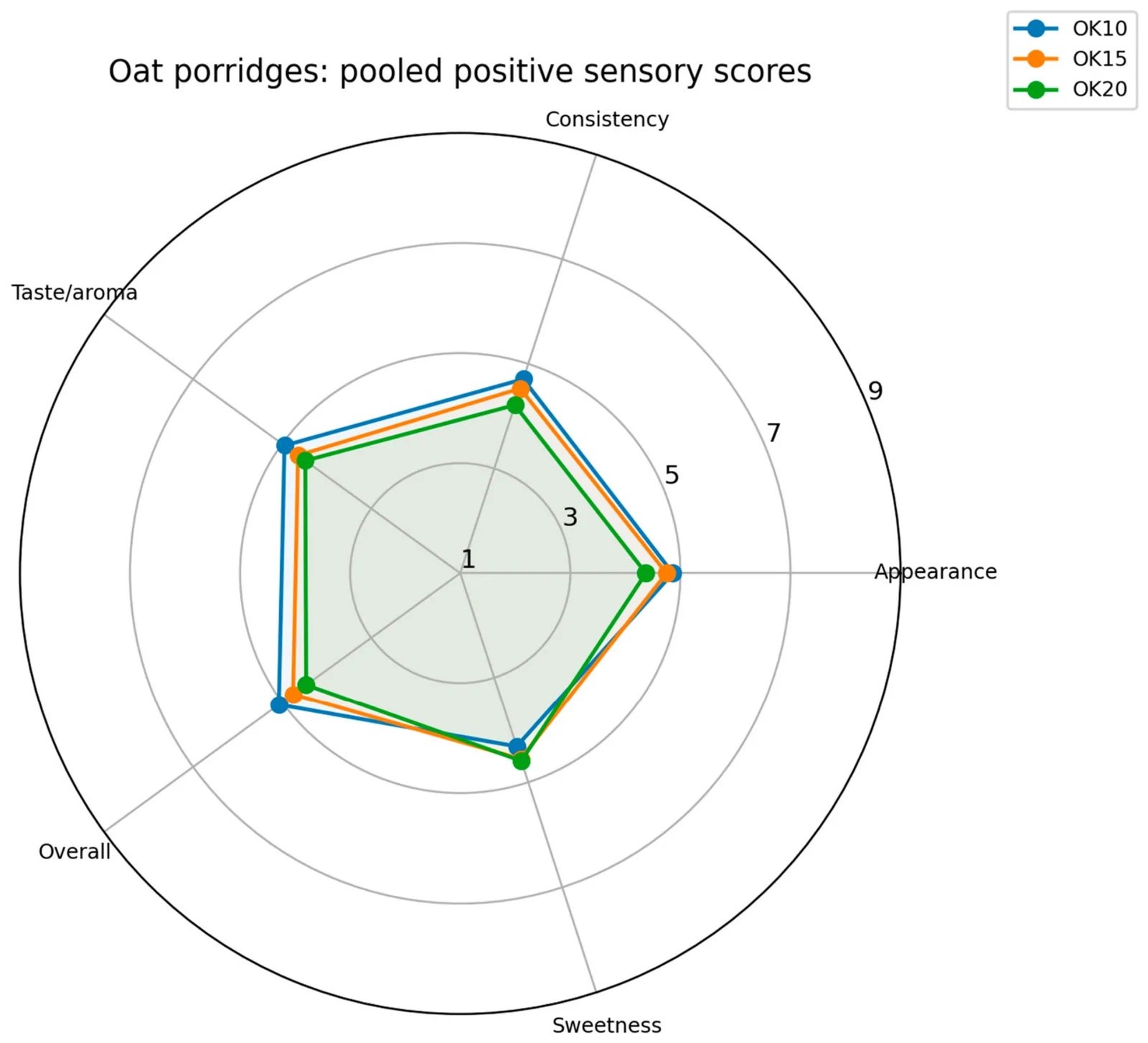
Pooled positive sensory radar plot of oat porridges showing appearance, consistency, taste and aroma, overall impression and sweetness. Values are pooled mean sensory scores across all available storage times and temperatures. The radar plot is descriptive and complements, but does not replace, the repeated-measures inferential analysis.

**Figure 8:**
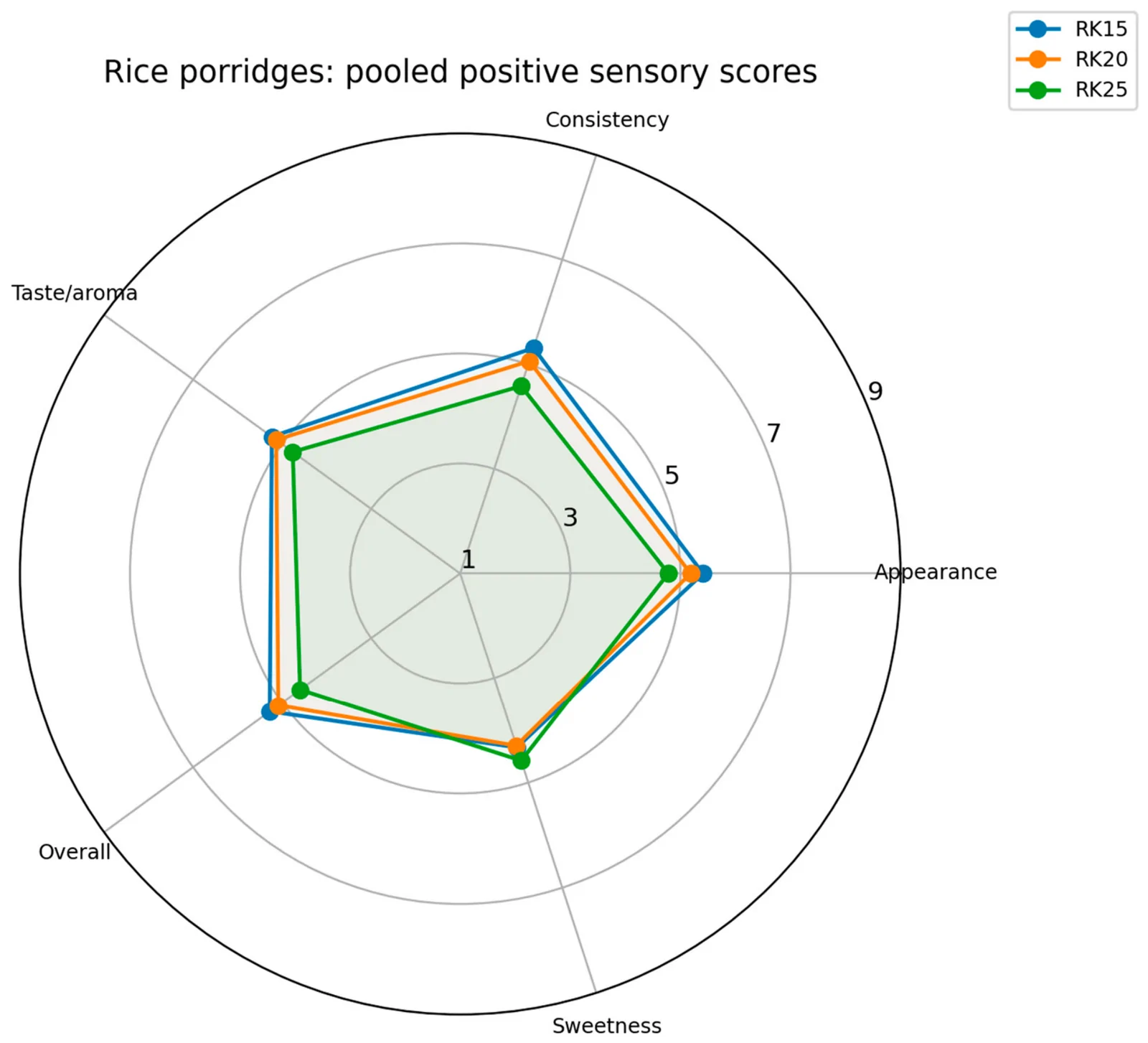
Pooled positive sensory radar plot of rice porridges showing appearance, consistency, taste and aroma, overall impression and sweetness. Values are pooled mean sensory scores across all available storage times and temperatures. The radar plot is descriptive and complements, but does not replace, the repeated-measures inferential analysis.

To complement the significance-based evaluation, pooled descriptive radar plots of the positive hedonic attributes (appearance, consistency, taste and aroma, overall impression and sweetness) were constructed separately for the oat and rice matrices (Figure 7 and Figure 8). These plots combine all available storage times and temperatures and therefore, provide formulation-level descriptive overviews rather than replacements for the session-specific repeated-measures inference. The radar plots are used only for descriptive visualization.

For oat porridges (Figure 7), the descriptive pooled positive means were highest for Oat10 regarding appearance (4.86), consistency (4.71), taste and aroma (4.95) and overall impression (5.07). Oat15 showed intermediate pooled values, while Oat20 had the lowest pooled values for these four positive attributes. These radar profiles are descriptive pooled summaries and do not establish superiority of any formulation.

For rice porridges (Figure 8), the descriptive pooled positive means were highest for Rice15: appearance, 5.41; consistency, 5.31; taste and aroma, 5.23; and overall impression, 5.28. Rice20 was intermediate, while Rice25 had the lowest pooled positive profile. These pooled profiles describe direction only and do not establish formulation superiority.

Table 7 provides the numerical basis for the pooled positive sensory radar plots. The corrected pooled means show the highest positive oat scores for Oat10 and the highest positive rice scores for Rice15. These values are descriptive summaries across available sessions and do not replace the repeated-measures inferential analysis.

### 3.8. Relationship Between Rheological Descriptors and Exploratory Sensory Response

The paired sensory–rheological dataset made it possible to test whether the instrumental description of rehydrated texture was reflected in sensory perception. Across all matched observations, the strongest and most interpretable associations were found for appearance, consistency and overall impression, i.e., the same attributes that are most directly influenced by the physical state of the rehydrated porridge. The overall correlations were moderate because the two cereal matrices did not behave in the same way; therefore, the matrix-separated analysis was more informative than the pooled analysis alone.

At the condition level, several unadjusted Spearman correlations were negative between apparent viscosity and positive sensory scores, particularly for rice porridges. [Table 8] For η40, rice correlations were ρ = −0.396 for appearance (*p* = 0.0031), ρ = −0.398 for consistency (*p* = 0.0029) and ρ = −0.344 for overall impression (*p* = 0.0109). In oat porridges, the corresponding η40 associations were weaker: appearance, ρ = −0.318 (*p* = 0.019); consistency, ρ = −0.247 (*p* = 0.072); and overall impression, ρ = −0.261 (*p* = 0.057). After adjustment for formulation, storage month and storage temperature in matrix-specific HC3 models, ln(η40) was not statistically distinguishable for any of the six primary sensory outcomes (all *p* ≥ 0.314). The same conclusion was obtained after exclusion of 9M. Accordingly, viscosity is described as co-varying with sensory scores in the unadjusted data, not as an independently demonstrated causal determinant.

### 3.9. Formulation-Level Rheological and Sensory Differences

Differences among the tested formulations were pronounced for rheology and were also visible in the exploratory sensory data. In the rice series, η40 increased from Rice15 through Rice20 to Rice25, while pooled positive sensory scores were lower in Rice25 than in Rice15. In the oat series, Oat20 showed the highest viscosity, whereas, among the three oat formulations, the corrected pooled positive sensory means were highest for Oat10. These contrasts cannot be attributed to cereal concentration alone because milk, sugar and other recipe components changed simultaneously. The results, therefore, identify formulation-level trade-offs rather than an experimentally determined optimum (Table 9).

The oat and rice series, therefore, show different combinations of instrumental rheology and exploratory sensory response. The corrected sensory mapping changes the descriptive oat ranking relative to the original analysis, but no formulation is interpreted as formally optimized. Mechanistic explanations for these differences are considered in Section 4.9.

The magnitude of formulation differences did not increase monotonically over storage time in the sensory dataset. Differences among formulations were present throughout storage, but their size fluctuated according to month, matrix and storage temperature. In contrast, the rheological dataset showed clearer temperature-dependent divergence. At 40 °C, formulation-related differences were larger than at 5 or 25 °C, especially for η40 and VRI η40. This indicates that accelerated storage amplified structural differences among formulations, even when sensory differences did not follow a simple linear ageing pattern.

### 3.10. Principal Component Analysis of Storage Stability and Rehydrated Quality

Principal component analysis (PCA) was used as an exploratory integrative tool. Both PCA models were recalculated from the final QC-checked balanced condition-level dataset (*n* = 108; 3–18 months at 5, 25 and 40 °C) [Table 10]. PCA1 included water activity, pH, moisture, dry-product ΔE, ln(η40), TVR and VRI η40. PCA2 included pH, rehydrated ΔE, ln(η40), TVR, VRI η40 and condition-level means for appearance, consistency, taste/aroma and overall impression. The underlying PCA analysis records contain input matrices, scores, loadings and explained-variance outputs and are retained by the authors; the main summaries are provided in Supplementary Tables S4–S7. PCA is interpreted as a description of covariance and clustering, not as evidence of causality or formulation optimization.

**Figure 9:**
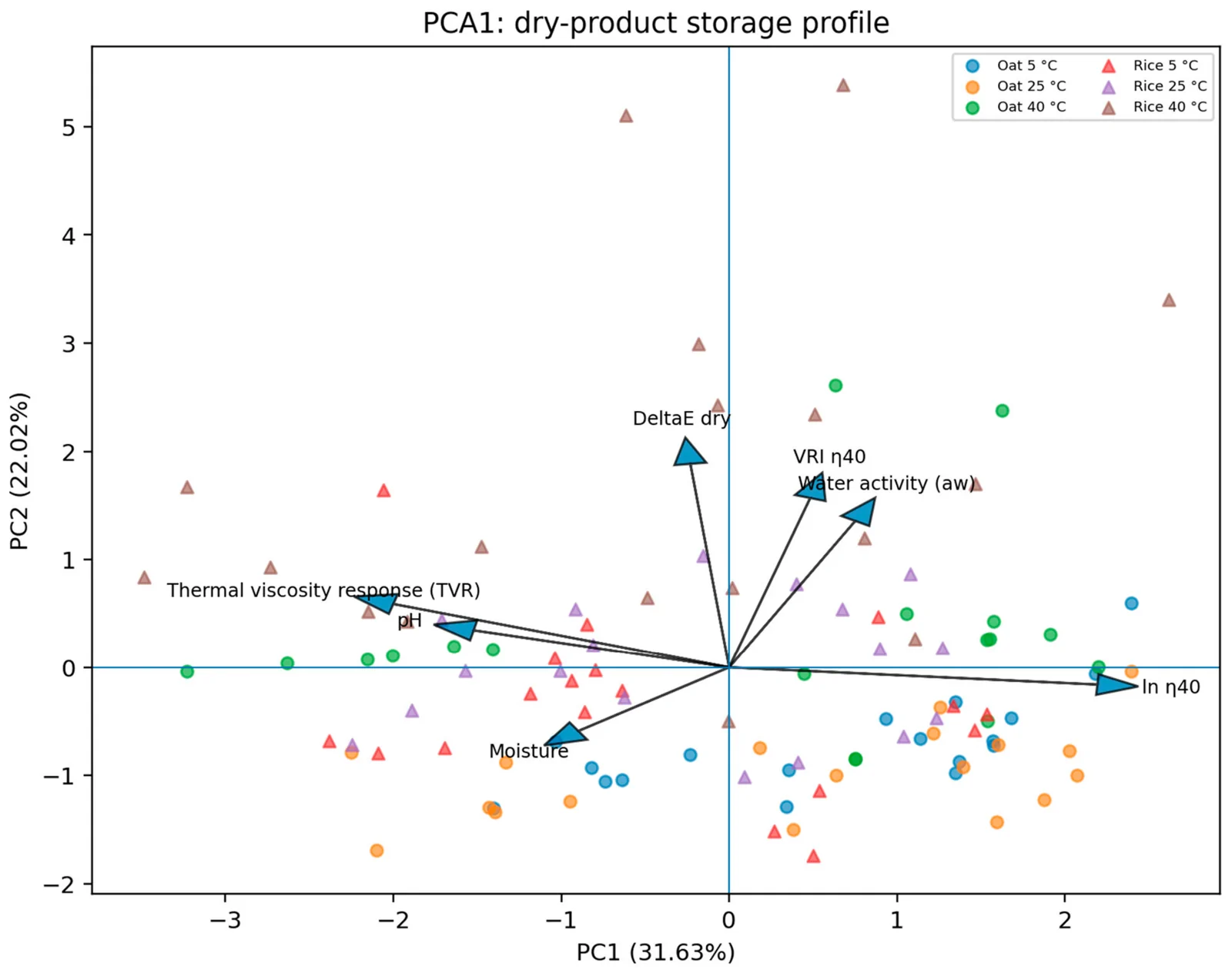
Revised PCA1 biplot describing the dry-product storage profile (*n* = 108 condition cells). The plot combines scores and loadings for water activity, pH, moisture, dry-product ΔE, ln(η40), TVR and VRI η40. Circles represent oat-based porridges, triangles represent rice-based porridges and fill colour indicates storage temperature. PCA is interpreted exploratorily as a covariance pattern.

In PCA1, PC1 and PC2 explained 31.63% and 22.02% of the variance [Figure 9], respectively (53.65% cumulative). The largest absolute correlation loadings on PC1 were ln(η40) (+0.90), TVR (−0.82) and pH (−0.65), while PC2 was most strongly associated with dry-product ΔE (+0.78), VRI η40 (+0.66) and water activity (+0.58). These loadings describe co-variation among measured descriptors and do not represent a stability ranking.

**Figure 10:**
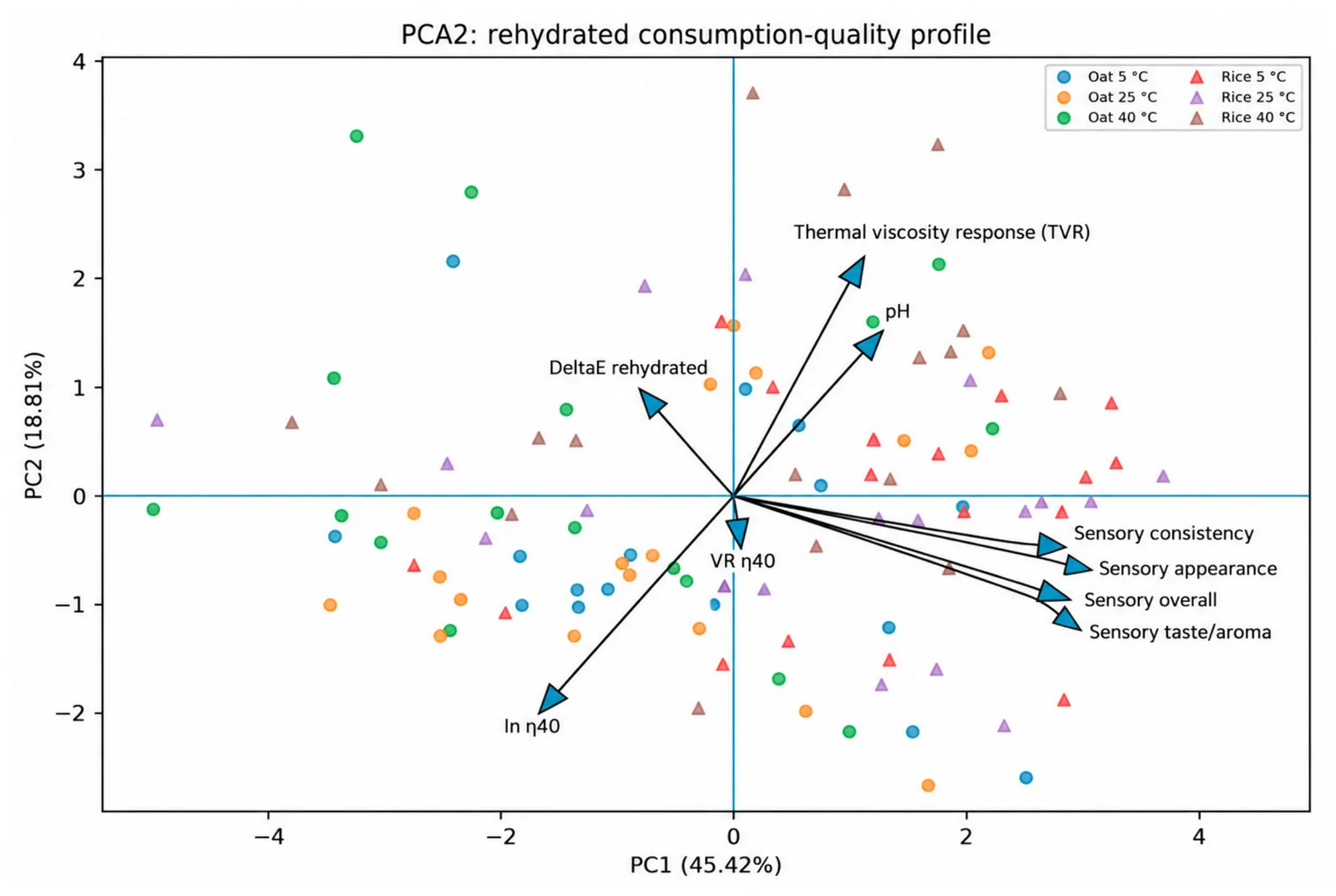
Revised PCA2 biplot describing the rehydrated consumption-quality profile (*n* = 108 condition cells). The plot combines pH, rehydrated ΔE, ln(η40), TVR, VRI η40 and the main sensory attributes. Circles represent oat-based porridges, triangles represent rice-based porridges and fill colour indicates storage temperature. Relative loading directions indicate covariance only and should not be interpreted as proof of causality or formulation optimality.

In PCA2, PC1 explained 45.42% and PC2 18.81% of the variance (64.23% cumulative), while PC3 explained an additional 13.32% (Figure 10). Appearance, consistency, taste/aroma and overall impression loaded strongly and positively on PC1 (correlation loadings +0.87 to +0.95), whereas ln(η40) loaded negatively (−0.59). TVR (+0.76), ln(η40) (−0.70) and pH (+0.53) contributed most strongly to PC2. Rehydrated ΔE had modest PC1/PC2 loadings but loaded more strongly on PC3 (+0.70), indicating a partially distinct colour-change dimension. Excluding 9-month observations produced highly similar PCA2 loading vectors (r = 0.999 for PC1, and r = 0.998 for PC2). These patterns are multivariate associations and do not establish causal sensory effects or an optimal formulation.

### 3.11. Microbiological Safety

Microbiological monitoring did not show progressive storage-related outgrowth across the monitored 0–18 month period. Quantified replicate counts were evaluated after individual log10(CFU/g) transformation, while threshold results were retained as censored observations rather than substituted by arbitrary numerical values. Isolated higher quantified replicates occurred, but they were not followed by monotonic increases during later storage and were interpreted together with the persistently low water activity(Table 11).

Yeast and mould results were predominantly at or below the reporting threshold, and Staphylococcus remained below the reporting threshold in the monitored rows. Enterobacteriaceae and aerobic sporulates showed sporadic quantified counts. Values originally reported as <10 or <100 CFU/g are presented as <1.00 or <2.00 log10(CFU/g), respectively. Mixed quantified/censored replicate sets are shown transparently and are not summarized by mean ± SD when the censoring pattern prevents a defensible numerical summary.

The microbiological interpretation is therefore based on storage trends, threshold-aware reporting and replicate-level variability rather than on arithmetic CFU/g means dominated by isolated observations. Detailed replicate-level results are retained in the checked microbiological dataset.

### 3.12. Chemical Composition and Nutritional Profile

Chemical composition analysis confirmed that all freeze-dried porridges were nutrient-dense, carbohydrate-rich dried meals, with matrix-dependent differences in protein, fat and saccharide fractions. At the initial analytical point, oat porridges contained approximately 15.75–16.66% protein, 12.62–15.55% fat, 66.74–70.81% saccharides and 0.84–1.06% salt. Rice porridges contained approximately 11.51–13.59% protein, 8.32–12.21% fat, 73.44–79.23% saccharides and 0.77–0.94% salt. Thus, the oat formulations generally provided higher protein and lipid fractions, whereas the rice formulations, particularly Rice20 and Rice25, were more carbohydrate-dominant. Baseline macronutrient composition is additionally visualized in Supplementary Figure S3, while the processed chemical-composition dataset is summarized in Supplementary Table S3.

The 12-month endpoint data did not suggest major deterioration of the bulk proximate composition during storage at 5, 25 or 40 °C. Across the available temperature-specific endpoint means [Table 12], oat porridges retained approximately the following levels: 15.69–16.73% protein, 12.64–16.26% fat, 65.69–70.62% saccharides and 1.00–1.51% salt. Rice porridges retained approximately 11.98–13.84% protein, 8.10–12.19% fat, 73.36–78.83% saccharides and 0.76–0.97% salt. Small differences among the initial and 12-month values should be interpreted with caution because the chemical dataset was available mainly at the baseline and endpoint rather than at all intermediate storage months. Nevertheless, the data indicate that macronutrient composition was not the primary shelf-life limitation in the present study; the most informative quality changes were rheological, visual and sensory rather than large shifts in protein, fat or saccharide content.

### 3.13. Integrated Interpretation of Shelf-Life Performance

The integrated 18-month endpoint at 40 °C is summarized in Table 13. This stress-condition overview combines safety-relevant, physicochemical, rheological and sensory descriptors and therefore, provides a compact practical snapshot of formulation performance under accelerated storage.

When all result domains are considered together, the tested formulations show different rheological, compositional and exploratory sensory profiles rather than a single demonstrably superior option. Oat20 had the highest oat viscosity, while the corrected pooled positive sensory means were highest for Oat10; Rice15 had the highest pooled positive rice scores, whereas Rice25 had the highest rice viscosity and lower pooled positive sensory scores. These comparisons are formulation-level observations because cereal, milk, sugar and other ingredients varied simultaneously. The chemical dataset adds a nutritional dimension, but it was measured at baseline and selected 12-month endpoints and therefore, does not establish continuous 18-month chemical stability.

The shelf-life performance of the freeze-dried porridges should therefore not be evaluated using a single parameter. Low aw and stable pH support safety, rheology describes practical rehydrated texture, colour reflects visible storage changes, and exploratory sensory testing indicates within-panel sensory response. Unadjusted condition-level viscosity–sensory associations were attenuated after adjustment for formulation, storage month and storage temperature, so the data do not establish viscosity as an independent causal determinant of sensory response. This multidimensional approach is consistent with modern ration development, where nutritional adequacy, physical stability and consumption behaviour must be considered together [1,12].

## 4. Discussion

### 4.1. Principal Findings and Position Within the Literature

The present study connects a practical ration-development problem with a mechanistic cereal-science question. Direct, factor-matched studies comparing freeze-dried oat and rice porridges are scarce, and the existing evidence is divided mainly among rice-porridge freeze-drying and rehydration studies, oat beta-glucan functionality studies, low-moisture food safety studies and military ration acceptability studies. The present dataset combines these streams in one design: oat versus rice matrix; formulation variants; high-barrier laminate packaging; storage at 5, 25 and 40 °C for up to 18 months; and simultaneous assessment of aw, moisture, colour, rheology, sensory quality, microbiology and chemical composition.

This integrated design is important because the shelf life of a freeze-dried ration meal is not determined by one endpoint. A product can remain microbiologically stable but become less suitable if the rehydrated texture becomes watery, sticky, unstable or visually unattractive. Conversely, a formulation with strong instrumental viscosity may be less suitable from a sensory-use perspective, depending on its within-panel sensory profile. The main contribution of the present study is the same-design comparison of the two cereal matrices across multiple shelf-life and rehydration-relevant domains, while recognizing that the observed differences are formulation-specific and do not establish a single optimal formulation.

### 4.2. Freeze-Drying, Rehydration and Matrix Structure

Freeze-drying usually preserves porous structure better than does hot-air drying, but the final rehydration behaviour depends strongly on the pre-freezing step, pore architecture and composition of the dried matrix. Using freeze-dried rice porridge, Rhim et al. [5] showed that freezing temperature can alter pore structure, friability, rehydration and water-vapour adsorption. This is directly relevant to the current products because hot-water preparation depends on rapid wetting, water penetration and reconstruction of a spoonable structure rather than on the properties of the dry product alone.

For oat matrices, freeze-drying and freezing can influence beta-glucan molecular weight, extractability and viscosity [6]. This provides a plausible literature-based framework for interpreting the oat products as hydrated biopolymer-particle networks rather than as simple cereal dispersions. After rehydration, the final texture may involve beta-glucan solubilization, starch swelling, protein and lipid interactions, and insoluble oat particles. This also explains why the sensory quality of oat porridges was not fully described by a single absolute viscosity value.

### 4.3. Matrix-Dependent Mechanisms: Oat Beta-Glucan Versus Rice Starch

The higher viscosity of oat porridges is consistent with the known functionality of oat beta-glucan. Oat beta-glucan viscosity depends on concentration, molecular weight and solubility, and processing can modify these properties [7,8,14,15]. In the present products, beta-glucans may have acted together with starch, proteins, lipids and insoluble fibre particles to contribute to the hydrated structure. Beta-glucan concentration, molecular weight and extractability were not measured, so this is a literature-supported mechanism rather than a directly demonstrated explanation for the higher η40 and η65 values observed at the common nominal formulation levels.

Rice matrices were more strongly governed by starch gelatinization, swelling and reorganization. Rice porridge rheology is known to be concentration-dependent and shear-thinning [20], while rice starch can undergo gelatinization, amylose leaching, retrogradation and reheating-induced structural changes [18,19]. The observed TVR values above 100% in several rice porridges are consistent with, but do not directly demonstrate, continued starch swelling or reorganization during the temperature ramp; TVR itself is not a measure of thermal stability.

Studies on rice starch mixed with beta-glucans or oat flour further support this interpretation. Different beta-glucans can modify rice-starch gelatinization and retrogradation [21]; oat beta-glucan can reduce retrogradation-related gel strengthening and syneresis [23]; and oat flour changes the pasting, rheological and tribological properties of rice-starch systems [22]. These studies do not reproduce the present product exactly, but they provide a strong mechanistic background for the matrix-dependent behaviour observed here.

### 4.4. Interpretation of VRI and TVR as Descriptive Indices

The literature review also highlights a methodological point: VRI and TVR are not standardized cereal-porridge descriptors. Most comparable cereal studies use raw apparent viscosity; RVA parameters such as peak, breakdown and setback; dynamic moduli or empirical texture tests. In this study, VRI and TVR are therefore treated as author-defined descriptive indices rather than as established universal parameters.

This does not weaken the analysis; it makes the interpretation more transparent. VRI summarizes the long-term change in apparent viscosity relative to the 0-month baseline, while TVR summarizes the temperature response during the rheological ramp. However, both indices must be reported together with η25, η40 and η65 because the same percentage value can arise from different absolute viscosity states. This is especially important for comparing oat and rice porridges: in oat products, a lower TVR may be consistent with thermal thinning, whereas in rice products, a higher TVR may be consistent with a net viscosity increase during the defined ramp; the underlying mechanism was not measured directly.

### 4.5. Water Activity, Moisture Migration and Microbiological Safety

The low aw values confirm the technological success of the drying and packaging strategy. Values around 0.07–0.13 were far below aw = 0.60, the practical region below which microbial growth is strongly restricted in low-moisture foods [9,11,12]. The current ISO framework for water activity measurement is ISO 18787:2017, which defines requirements for determining aw in foods and feeds within the 0–1 range [36]. The low-aw state was therefore a meaningful safety and stability indicator, but it should not be interpreted as a sterilization step.

The microbiological results support this distinction. The absence of systematic increases in TVC, yeasts, moulds, staphylococci, Enterobacteriaceae and aerobic sporulates is consistent with the absence of progressive microbial outgrowth under the tested combination of drying, hygienic processing and high-barrier packaging. Sporadic countable values, including aerobic sporulates, are better interpreted as residual raw-material or process-related background contamination than as shelf-life-limiting growth because they were not accompanied by progressive increases over time or by increased aw. This is consistent with the low-moisture food literature, which emphasizes that microorganisms can survive under dry conditions, even when they cannot proliferate [10,11].

Moisture migration remains important, even when aw is low. Water activity is thermodynamically linked to equilibrium relative humidity, so any loss of package integrity or exposure to humid environments can lead to moisture uptake, structural plasticization and changes in texture. Oat storage studies show that temperature and relative humidity strongly influence water migration and sorption behaviour [41]. Similarly, storage studies on freeze-dried fruit-based snacks demonstrate that aw, water content, texture and colour can fluctuate during 4/25/40 °C storage, even in barrier packaging, without necessarily exceeding microbial safety limits [13]. The present data fit this broader pattern: microbiological safety was maintained, while the more informative shelf-life changes were physicochemical, rheological and sensory.

### 4.6. Colour, Oxidative Stability and Sensory Drift

Colour change was one of the most visible indicators of product evolution. In fruit-containing cereal systems, changes in L*, a*, b* and ΔE can arise from pigment degradation, non-enzymatic browning, moisture-related changes in light scattering and matrix structural changes [13,26,42]. However, ΔE should be interpreted carefully. De Flaviis and Sacchetti [27] note that food studies often transfer general ΔE perception thresholds without matrix-specific validation. Therefore, the present manuscript should treat ΔE primarily as an objective marker of visual drift, not as definitive evidence of sensory rejection.

Oat formulations require an additional shelf-life perspective because they contain more lipid-rich cereal material than do rice formulations. Long-term oat storage studies have linked lipid oxidation and hydrolytic changes to sensory descriptors such as earthy, musty and rancid notes [16], and recent oat-flour work confirms the importance of hydrolytic and oxidative rancidification during 12-month storage [17]. In the present study, rancidity-related descriptors did not appear to be the primary limiting factor compared with texture-related attributes, but oxidative stability should remain a priority in future work, especially for oat- and milk-containing formulations stored under warm conditions. However, peroxide value, secondary lipid-oxidation markers, volatile oxidation products and headspace oxygen were not measured. Low water activity and microbiological stability, therefore, cannot be interpreted as proof of complete oxidative stability over the entire storage period, particularly for the oat- and milk-containing formulations.

### 4.7. Coupling Between Instrumental Rheology and Exploratory Sensory Response

The unadjusted condition-level data showed several negative associations between apparent viscosity and positive sensory scores, especially in the rice matrix. These associations were attenuated after adjustment for formulation, storage month and storage temperature, and ln(η40) was not independently significant in any of the six primary models. The most defensible interpretation is therefore that viscosity and exploratory sensory response co-varied with broader formulation and storage effects; the analysis does not establish a direct sensory penalty or benefit of viscosity itself.

The matrix-specific crude patterns can still be considered in the context of published cereal rheology, but any explanation involving starch swelling, beta-glucan functionality or structural coherence remains a mechanistic interpretation rather than a direct result of the adjusted sensory–rheology models.

The pooled radar plots are descriptive visual summaries only. The corrected oat mapping shows the highest pooled positive means for Oat10, while Rice15 has the highest pooled positive rice scores. These radar profiles do not replace the repeated-measures inferential analysis and do not demonstrate formulation superiority.

### 4.8. Multivariate Description of Quality Domains

The recalculated PCA models provide exploratory multivariate summaries of the integrated dataset. PCA1 and PCA2 used the balanced *n* = 108 condition matrix and avoided simultaneous entry of multiple highly collinear viscosity variables. PCA1 described co-variation among ln(η40), TVR, dry-product colour drift, VRI, aw, pH and moisture. PCA2 showed a strong positive sensory-loading pattern opposed to ln(η40) on PC1, while rehydrated ΔE contributed more strongly to PC3. These loading patterns describe covariance and sample positioning; they do not determine formulation optimality or causality. The near-identical PCA2 loading structure after exclusion of 9M supports the robustness of the main multivariate pattern.

### 4.9. Formulation-Level Differences as a Design Variable

The formulation series should be interpreted as complete recipe variants rather than as an isolated cereal-concentration experiment. Increasing the nominal cereal label was accompanied by changes in milk, sugar and other ingredients, so the observed rheological and exploratory sensory differences cannot be assigned uniquely to cereal concentration. In the rice series, Rice15 had the lowest viscosity and the highest corrected pooled positive scores, while Rice25 had the highest viscosity and lower pooled positive scores. These patterns are relevant to formulation development but do not constitute formal optimization.

The oat series likewise showed formulation-level trade-offs. Oat20 had the highest apparent viscosity, whereas the corrected pooled positive sensory means were highest for Oat10 and intermediate for Oat15. Literature on beta-glucan extractability, molecular weight and processing history provides possible mechanistic context [6,7,8], but beta-glucan properties were not measured directly in the present study.

The fact that formulation differences were amplified at 40 °C is important for military and emergency logistics. Elevated temperature did not simply reduce quality uniformly; rather, it exposed differences among formulations. Accelerated dry-heat storage can therefore be useful for screening unstable textural trajectories, but it is not a perfect surrogate for hot and humid field environments. Future shelf-life protocols should combine temperature stress with controlled humidity or package-challenge tests.

### 4.10. Nutritional and Chemical Composition in the Context of Ration Design

The proximate composition data support the intended role of the products as energy-dense breakfast or light-meal ration components rather than as complete stand-alone daily rations. The predominance of saccharides is technologically and nutritionally expected for cereal-based porridges and is operationally relevant because carbohydrate-rich warm meals can provide rapidly usable energy in field conditions. At the same time, the matrix-dependent differences are important for ration planning: oat formulations supplied comparatively higher protein and lipid fractions, while rice formulations were more carbohydrate-dominant and lower in fat, particularly in the higher nominal rice-level formulations.

No major change in bulk proximate composition was observed between baseline and the measured 12-month endpoint under the tested packaging and storage conditions. This does not exclude oxidative or sensory changes, especially in oat- and milk-containing formulations, where lipid oxidation can influence flavour before large changes in total fat content are analytically apparent. Therefore, nutritional composition should be interpreted together with sensory descriptors, colour, microbiology and, where possible in future work, specific markers of lipid oxidation or protein modification.

### 4.11. Practical Relevance for Military and Emergency Rations

The freeze-dried porridge concept is relevant for military and emergency feeding because the products are lightweight, compact, rapidly rehydratable and suitable for breakfast or light-meal use. Such meals may be valuable when users need a warm, easily digestible, carbohydrate-rich product rather than a heavy main dish. Ration literature indicates that palatability, monotony, meal context and ease of preparation strongly affect actual intake [1,2,3].

The present study also fits the practical logic of operational ration standards, where shelf life, ease of preparation, nutrient delivery and field usability must be considered together [32]. The analytical domains used here map well onto that logic: aw and microbiology indicate safety; moisture and packaging indicate barrier performance; rheology indicates preparation-relevant texture; colour indicates visual quality; and exploratory sensory analysis captures user-relevant sensory response. A ration component can be microbiologically stable but fail operationally if it is difficult to rehydrate or unpleasant to consume.

Because the final analysed sensory panel was small, the sensory outcomes should be framed as exploratory operational screening rather than broad consumer acceptance. ISO 11136:2014 [28] provides guidance for hedonic tests in controlled areas, and Hough et al. [43] showed that substantially larger panels are usually needed for robust consumer acceptability conclusions. The present sensory results are nevertheless valuable as controlled ration-development data because they were obtained from a consistent harmonized panel and were linked to objective rheological descriptors. The repeated-measures inferential dataset was restricted to the same eight longitudinal assessors; additional students who participated only in selected sessions were not included in the longitudinal inference.

### 4.12. Limitations and Recommendations

Several limitations should be considered when interpreting the results. Microbiological reporting thresholds and acceptance criteria depend on the target regulatory or internal military-ration specification; therefore, safety interpretation should be aligned with the specification used for deployment or submission. Previously erroneous moisture values were recalculated from the original gravimetric records, with raw records and corrections retained in the QC log. The inferential sensory analysis was restricted to the same longitudinal panel of eight assessors; additional assessors who participated only in selected sessions were excluded. All replicate pouches originated from the same production batch, so replication was achieved at the packaged storage-unit level rather than at the independent production-batch level.

Additional limitations are interpretative. VRI and TVR are author-defined indices and are presented together with absolute η25, η40 and η65 values. Unadjusted sensory–rheological associations are exploratory, and the adjusted models did not establish an independent effect of ln(η40) after formulation, storage month and storage temperature were considered. Colour differences are interpreted as objective ΔE*ab markers rather than direct sensory rejection thresholds. Chemical composition data were available mainly for the baseline and 12-month endpoint; therefore, they support formulation verification and endpoint stability interpretation rather than complete kinetic modelling of chemical composition. Peroxide value, secondary oxidation products, volatile oxidation markers and headspace oxygen were not measured, which limits conclusions regarding oxidative stability. Beta-glucan concentration, molecular weight and extractability were also not determined directly.

Future work should include moisture sorption isotherms, oxidative stability markers for oat- and milk-containing formulations, headspace oxygen monitoring, and preparation trials under field-like conditions. Further sensory work should involve larger military-user panels and direct comparison of serving temperatures, water addition levels and waiting times. A useful follow-up would be a targeted validation of the observed formulation-level trade-offs, including direct measurement of portion consumption under realistic preparation constraints.

## 5. Conclusions

Freeze-dried oat- and rice-based porridges packed in aluminium multilayer laminate pouches showed potential under the investigated conditions as lightweight, shelf-stable components for military, emergency and humanitarian rations. The products maintained very low water activity, stable pH and satisfactory microbiological status during storage, indicating that microbial growth was not the primary shelf-life limitation under the tested conditions. Chemical composition confirmed carbohydrate-rich profiles, with oat formulations generally providing higher protein and lipid fractions and rice formulations showing a more carbohydrate-dominant profile; 12-month endpoint data did not indicate major macronutrient drift. The most informative quality changes were therefore matrix-dependent rheological and exploratory sensory changes after rehydration. Oat formulations showed higher apparent viscosity than did rice formulations at comparable nominal formulation levels. Beta-glucan functionality is one plausible literature-supported explanation, but beta-glucan concentration, molecular weight and extractability were not measured directly in this study. Rice porridges had lower absolute viscosity but stronger temperature-dependent thickening, consistent with starch swelling and reorganization. Unadjusted sensory–rheology associations were attenuated after adjustment for formulation, storage month and storage temperature; the analysis, therefore, does not establish an independent sensory effect of viscosity or identify an optimal formulation. Corrected pooled positive scores were highest for Oat10, within the oat series, and Rice15, within the rice series, but these pooled values are descriptive. PCA described related but distinct multivariate quality patterns and is interpreted exploratorily rather than as a method for formulation optimization. Overall, freeze-dried porridge development for operational rations should combine low-aw safety, barrier packaging, matrix-specific rheology, colour stability, microbiology and exploratory sensory quality in a single shelf-life framework.

## Figures and Tables

**Table 1:** Oat porridge formulations.

Variant	Oat Flakes (g)	Whole Milk (g)	Sugar (g)	Salt (g)	Strawberries (g)
Oat10	3000	22,000	425	15	2840
Oat15	3250	16,000	310	10	2065
Oat20	4500	15,500	300	10	2000

Note: Values are given as prepared batch masses. The numerical variant code denotes the nominal cereal level used for formulation labelling.

**Table 2:** Rice porridge formulations.

Variant	Rice (g)	Whole Milk (g)	Sugar (g)	Salt (g)	Strawberries (g)
Rice15	3200	15,000	445	15	2675
Rice20	3700	12,000	355	12	2140
Rice25	4060	10,100	300	10	1800

Note: Values are given as prepared batch masses.

**Table 3:** Physicochemical ranges used for stability interpretation.

Matrix	Parameter	Approximate Range in Lyophilized Samples	Interpretation
Oat porridges	aw	0.074–0.133	Very low water availability; supportive of microbiological stability
Rice porridges	aw	0.067–0.165	Very low water availability; RK20 1M value corrected to 0.0846 ± 0.0011 (*n* = 3)
Oat porridges	pH	5.730–6.057	Stable, slightly acidic to near-neutral profile
Rice porridges	pH	5.717–6.070	Stable, slightly acidic to near-neutral profile
Oat porridges	Moisture	0.717–2.910%	Low residual moisture after recalculation from original gravimetric records
Rice porridges	Moisture	1.248–2.650%	Low residual moisture after recalculation from original gravimetric records

Note: Ranges exclude fresh hydrated products. Previously flagged moisture rows were recalculated from the original gravimetric records, as described in Section 2.7.3; sample SDs were recalculated consistently, and the raw records were retained in the QC log; the RK20 1M water-activity entry was author-corrected to 0.0846 ± 0.0011 (*n* = 3).

**Table 4:** Key rheological parameters after 18 months of storage.

Variant	Storage (°C)	n_tech	η40 (mPa·s)	η65 (mPa·s)	VRI η40 (%)	VRI η65 (%)	TVR (%)
Oat10	5	4	4434 ± 393	3278 ± 189	112.8	99.3	99.8
Oat10	25	4	2959 ± 268	4232 ± 164	75.3	128.1	156.3
Oat10	40	4	1882 ± 167	3169 ± 226	47.9	96.0	167.9
Oat15	5	4	7901 ± 435	5913 ± 358	110.3	124.2	48.3
Oat15	25	4	7641 ± 604	5665 ± 426	106.7	119.0	47.3
Oat15	40	4	11,228 ± 1007	7727 ± 556	156.8	162.3	83.6
Oat20	5	4	13,471 ± 564	9807 ± 277	79.6	83.7	44.6
Oat20	25	3	15,503 ± 766	11,893 ± 946	91.6	101.6	46.8
Oat20	40	3	17,294 ± 926	12,580 ± 108	102.1	107.4	44.4
Rice15	5	3	1604 ± 58	2080 ± 65	131.8	104.8	101.7
Rice15	25	4	1607 ± 115	2300 ± 166	132.0	115.9	117.8
Rice15	40	3	1266 ± 120	2945 ± 109	104.1	148.4	210.9
Rice20	5	4	2889 ± 65	4599 ± 283	109.3	103.4	125.5
Rice20	25	4	5079 ± 207	5414 ± 81	192.1	121.7	75.8
Rice20	40	4	6595 ± 1023	8095 ± 148	249.4	181.9	132.5
Rice25	5	6	7746 ± 1082	7532 ± 1012	98.3	81.2	71.9
Rice25	25	4	9750 ± 449	10,468 ± 598	123.7	112.8	100.3
Rice25	40	4	13,919 ± 1765	13,192 ± 1219	176.7	142.2	121.5

Note: η40 and η65 are apparent viscosities extracted during the temperature ramp. VRI is calculated relative to the corresponding 0M baseline. TVR = η65/η25 × 100; values above 100% indicate only that η65 exceeded η25 under the defined ramp. Here, n_tech denotes the number of technical rheological runs performed on a single composite prepared by combining two stored pouches from the same condition; the reported SD, therefore, describes technical repeatability rather than independent storage-unit variability.

**Table 5:** Condition-level statistical comparison of oat and rice matrices at common 15 and 20% formulations during 3–18 months.

Metric	n_cond Oat	Mean Oat	n_cond Rice	Mean Rice	Oat/Rice Ratio	*p* Value	Hedges’ g
η25 (mPa·s)	36	17,780	36	3403	5.22	<0.001	2.70
η40 (mPa·s)	36	11,902	36	2899	4.11	<0.001	3.06
η65 (mPa·s)	36	8581	36	4024	2.13	<0.001	1.96
TVR (%)	36	51.1	36	131.7	0.39	<0.001	−2.78
VRI η40 (%)	36	104.5	36	143.9	0.73	<0.001	−1.14
VRI η65 (%)	36	111.2	36	123.7	0.90	0.016	−0.57

Welch tests were recalculated using condition-level composite means. Each matrix contributed 36 condition-level values (2 common nominal formulation levels × 6 storage months × 3 storage temperatures). For each condition, material from two independently stored pouches was combined, and ≥3 technical rheological runs were averaged before analysis. Technical runs were, therefore, not treated as independent observations. Hedges’ g was calculated from the 36 condition-level values per matrix.

**Table 6:** Summary of sensory statistical outcomes.

Area	Revised Result
Primary omnibus analysis	560 Friedman tests; 224 raw *p* < 0.05 (40.0%)
Multiplicity sensitivity	116/560 omnibus tests retained under global BH-FDR sensitivity analysis
Post hoc analysis	Paired Wilcoxon signed-rank tests with Holm adjustment after significant Friedman tests
Significant pairwise contrasts	154 Holm-adjusted formulation contrasts
Matrix summary	Oat: 120/280 raw significant; rice: 104/280 raw significant; main positive attributes remained most robust

Note: Higher ranks indicate higher original scores. For positive hedonic attributes, this generally means better perception; for negative intensity descriptors, it means greater perceived intensity and is not automatically favourable. The formulation code mapping used for the integrated analysis was harmonized with the final Oat10/Oat15/Oat20 and Rice15/Rice20/Rice25 nomenclature.

**Table 7:** Pooled descriptive sensory scores for the main positive attributes of the harmonized formulations.

Formulation	Appearance	Consistency	Taste and Aroma	Overall Impression	Sweetness
Oat10	4.86	4.71	4.95	5.07	4.32
Oat15	4.75	4.53	4.64	4.75	4.56
Oat20	4.37	4.21	4.48	4.46	4.59
Rice15	5.41	5.31	5.23	5.28	4.33
Rice20	5.19	5.06	5.13	5.09	4.30
Rice25	4.78	4.58	4.76	4.60	4.58

Note: Values are pooled means across all available storage times and temperatures. Higher values indicate higher recorded scores within the applied scale. These pooled means are descriptive and should not be interpreted as evidence of formulation superiority.

**Table 8:** Condition-level unadjusted η40 associations and primary adjusted sensory–rheology models.

Matrix	Outcome	*n*	Unadjusted η40 ρ (*p*)	Adjusted β ln(η40) (95% CI)	HC3 p	Adjusted R^2^
Oat	Appearance	54	−0.318 (0.019)	+0.306 (−0.937 to 1.549)	0.622	0.229
Oat	Consistency	54	−0.247 (0.072)	+0.394 (−1.139 to 1.926)	0.607	0.174
Oat	Overall impression	54	−0.261 (0.057)	+0.365 (−1.083 to 1.813)	0.614	0.306
Rice	Appearance	54	−0.396 (0.0031)	−0.039 (−0.696 to 0.618)	0.905	0.545
Rice	Consistency	54	−0.398 (0.0029)	−0.416 (−1.240 to 0.408)	0.314	0.456
Rice	Overall impression	54	−0.344 (0.0109)	−0.026 (−1.079 to 1.027)	0.961	0.345

Note: Unadjusted Spearman coefficients are exploratory condition-level associations. Adjusted models used *n* = 54 condition cells per matrix and included categorical formulation, storage month and storage temperature; HC3 robust standard errors were used. The adjusted ln(η40) term was not statistically significant in any of the six primary models.

**Table 9:** Pooled formulation-level sensory and rheological profile of the harmonized formulations.

Matrix	Form.	Overall Impression	Consistency	Appearance	η40 (mPa·s)	η65 (mPa·s)	Interpretation
Oat	Oat10	5.10	4.72	4.83	2699	3618	Lowest oat viscosity; highest corrected pooled positive oat scores in the balanced window
Oat	Oat15	4.76	4.40	4.64	8524	6115	Intermediate oat rheology and sensory profile
Oat	Oat20	4.56	4.22	4.41	15,323	11,070	Highest oat viscosity; lower pooled positive scores
Rice	Rice15	5.27	5.30	5.46	1547	2376	Lowest rice viscosity; highest pooled positive rice scores
Rice	Rice20	5.14	5.14	5.28	4193	5661	Intermediate rice profile
Rice	Rice25	4.55	4.48	4.68	9604	10,027	Highest rice viscosity; lower pooled positive scores

Note: Values are pooled means from the matched sensory–rheological dataset. The table is intended for formulation-level interpretation rather than as a replacement for the full storage-time and storage-temperature tables. η40 and η65 are apparent viscosities extracted at approximately 40 and 65 °C during the temperature ramp.

**Table 10:** Revised summary of PCA models and explained variance.

Model	Purpose	*n*	PC1 (%)	PC2 (%)	PC1 + PC2 (%)	PC3 (%)
PCA1	Dry-product storage profile	108	31.63	22.02	53.65	14.29
PCA2	Rehydrated consumption-quality profile	108	45.42	18.81	64.23	13.32

**Table 11:** Threshold-aware log-scale summary of microbiological findings during storage.

Indicator	Data Presentation	Overall Storage Interpretation
Total viable count	Quantified replicates as log10(CFU/g); censored values retained at source threshold	Mostly low; isolated higher replicates did not show progressive outgrowth
Yeasts	<2.00 log10(CFU/g) retained where reported as <100 CFU/g	Predominantly below threshold; sporadic quantified observations
Moulds/fungi	<2.00 log10(CFU/g) retained where reported as <100 CFU/g	Predominantly below threshold; no sustained increase
Staphylococcus	Threshold results retained without substitution	Below reporting threshold in monitored rows
Enterobacteriaceae	Quantified values shown on log scale; <10 retained as <1.00 log10(CFU/g)	Sporadic low counts; no systematic increase
Aerobic sporulates	Quantified values on log scale; threshold observations preserved	Low-to-moderate resistant-spore background; no progressive outgrowth

Note: Positive numeric replicates were transformed individually as log10(CFU/g). Reporting-threshold observations were retained as censored values and were not replaced by arbitrary values, such as half the reporting limit. Mixed quantified/censored replicate sets are displayed transparently rather than forced into numerical mean ± SD summaries.

**Table 12:** Chemical composition of freeze-dried porridge formulations at baseline and after 12 months of storage.

Formulation	Initial Composition, 0 M (% of Lyophilized Product)	12-Month Endpoint Across 5/25/40 °C (%; Range of Temperature-Specific Means)	Interpretation
Oat10	Protein 16.66 +/− 0.10	Protein 16.58–16.73	Highest oat fat-fraction and lowest oat saccharide-fraction; composition remained broadly stable at 12 months.
	Fat 15.54 +/− 0.06	Fat 15.63–16.25	
	Saccharides 66.74 +/− 0.00	Saccharides 65.69–66.28	
	Salt 1.06 +/− 0.04	Salt 1.36–1.51	
Oat15	Protein 16.16 +/− 0.03	Protein 15.85–16.17	Balanced oat formulation with intermediate protein/fat/saccharide profile.
	Fat 14.21 +/− 0.08	Fat 14.54–14.88	
	Saccharides 68.72 +/− 0.11	Saccharides 67.98–68.47	
	Salt 0.91 +/− 0.00	Salt 1.00–1.21	
Oat20	Protein 15.75 +/− 0.02	Protein 15.68–15.86	Most carbohydrate-rich oat formulation and lowest oat fat-fraction; endpoint values remained close to baseline.
	Fat 12.62 +/− 0.06	Fat 12.64–13.47	
	Saccharides 70.81 +/− 0.02	Saccharides 69.66–70.62	
	Salt 0.83 +/− 0.02	Salt 1.06–1.12	
Rice15	Protein 13.59 +/− 0.05	Protein 13.66–13.84	Rice formulation with the highest protein and fat fractions within the rice set.
	Fat 12.21 +/− 0.14	Fat 11.59–12.18	
	Saccharides 73.44 +/− 0.19	Saccharides 73.36–73.80	
	Salt 0.77 +/− 0.00	Salt 0.77–0.80	
Rice20	Protein 11.75 +/− 0.15	Protein 12.00–12.46	Intermediate rice formulation; more carbohydrate-dominant than Rice15.
	Fat 9.09 +/− 0.03	Fat 9.25–9.46	
	Saccharides 78.37 +/− 0.22	Saccharides 77.26–77.93	
	Salt 0.80 +/− 0.04	Salt 0.76–0.82	
Rice25	Protein 11.51 +/− 0.03	Protein 11.97–12.23	Most carbohydrate-rich rice formulation and lowest fat fraction; endpoint values remained close to baseline.
	Fat 8.32 +/− 0.23	Fat 8.09–8.46	
	Saccharides 79.23 +/− 0.20	Saccharides 78.58–78.83	
	Salt 0.94 +/− 0.00	Salt 0.94–0.97	

Note: Baseline values are mean +/− SD from available analytical replicates. The 12-month endpoint is presented as the range of temperature-specific means across 5, 25 and 40 °C. Intermediate storage months were not fully populated in the updated chemical worksheet; therefore, this table supports formulation and endpoint stability interpretation rather than complete kinetic modelling of chemical composition.

**Table 13:** Integrated quality profile of freeze-dried porridge formulations after 18 months of storage at 40 °C.

Formulation	aw	Moisture (%)	pH	ΔE*ab	η40 (mPa·s)	η65 (mPa·s)	TVR (%)	Overall Impression
Oat10	0.081	2.26	5.91	5.97	1883	3169	167.9	5.25
Oat15	0.133	1.69	5.91	3.85	11,229	7727	83.6	4.12
Oat20	0.084	1.31	5.87	4.52	17,299	12,580	44.7	3.75
Rice15	0.075	1.74	6.05	7.18	1266	2945	212.1	4.62
Rice20	0.080	1.82	5.96	6.63	6595	8095	132.5	3.88
Rice25	0.083	1.53	5.78	5.80	13,919	13,192	121.5	3.38

Note: aw, moisture, pH and ΔE*ab refer to lyophilized products. η40, η65 and TVR were extracted from the temperature-ramp rheological analysis. Overall impression represents the mean sensory score at 18 months and 40 °C. Previously flagged moisture entries were recalculated from the original gravimetric records, as described in Section 2.7.3, with the raw records retained for traceability.

## Data Availability

The original contributions presented in this study are included in the article and Supplementary Material. Further inquiries can be directed to the corresponding author.

## Supplementary Materials

The following supporting information can be downloaded at: https://www.mdpi.com/article/10.3390/foods15213829/s1, Table S1: Overview of supplementary figures and tables; Table S2: Summary of experimental design and analytical domains; Table S3: Overview of processed datasets retained by the authors; Figure S1: Extended trends of selected physicochemical stability markers during storage; Figure S2: Rheological endpoint after 18 months of storage at 40 °C; Figure S3: Baseline macronutrient composition of freeze-dried porridge formulations at 0M; Tables S4–S7: PCA loadings and explained-variance summaries.
