# Supplementary material for: Freeze-Dried Oat- and Rice-Based Porridges for Military and Emergency Rations: Formulation, Long-Term Storage Stability, Rheological Behaviour and Exploratory Sensory Evaluation
Source: Foods. 2026 Oct 26;15(21):3829. doi: 10.3390/foods15213829 (PMC13762730; doi:10.3390/foods15213829)
Supplement: Supplementary file 1 [file foods-15-03829-s001.zip › foods-4610810-supplementary.pdf]

Freeze-dried oat- and rice-based porridges for military and emergency rations: formulation, long-term storage stability, rheological behaviour and exploratory sensory evaluation

Martin Szotkowski 1,*; Jiri Malisek 1; Kristina Trenzova 1; Katerina Kristofova 1,2; Monika Hartmannova 3; Blanka Dostalova 3

1 Laboratory of Food Quality and Safety Research, Department of Logistics, Faculty of Military Leadership, University of Defence, Kounicova 65, 662 10 Brno, Czech Republic

2 Department of Plant Origin Food Sciences, Faculty of Veterinary Hygiene and Ecology, University of Veterinary Sciences, Palackeho tr. 1946/1, 612 42 Brno, Czech Republic

3 Central Military Veterinary Institute, Ministry of Defence, Opavska 771/29, 748 01 Hlucin, Czech Republic

*Correspondence: martin.szotkowski@unob.cz

This supplementary file provides only additional figures, tables and model outputs that are not reproduced in the main manuscript. The supplementary material is organized to provide traceability for the broader processed dataset, the analytical design and the extended PCA outputs.

Contents of supplementary material

Table S1. Overview of the supplementary figures and tables included in this supplementary file.

Item	Content
Table S1	Overview of the supplementary figures and tables included in this supplementary file.
Table S2	Summary of the experimental design and analytical domains.
Table S3	Overview of processed datasets retained by the authors.
Figure S1	Extended trends of selected physicochemical stability markers during storage.
Figure S2	Rheological endpoint after 18 months of storage at 40 °C (η40 and η65).
Figure S3	Baseline macronutrient composition of freeze-dried porridge formulations at 0M.
Table S4	PCA1 loadings for the first two principal components.
Table S5	PCA2 loadings for the first two principal components.
Table S6	PCA1 explained variance for the first five components.
Table S7	PCA2 explained variance for the first five components.

Supplementary study design and processed datasets

Table S2. Summary of the experimental design and analytical domains.

Domain	Description
Product matrices	Oat-based freeze-dried porridges; Rice-based freeze-dried porridges

Formulations	Oat10, Oat15, Oat20; Rice15, Rice20, Rice25
Storage temperatures	5 °C, 25 °C, 40 °C
Storage times	0M, 1M, 3M, 6M, 9M, 12M, 15M, 18M (depending on analysis domain)
Physicochemical analyses	Water activity, pH, moisture content, colour coordinates and ΔE*ab
Rheological analyses	Apparent viscosity (η25, η40, η65), thermal viscosity response ratio (TVR) and viscosity retention indices For each condition, reported within-condition n values refer to technical runs of the two-pouch composite.
Sensory analyses	Appearance, consistency, taste and aroma, overall impression, sweetness and defect/intensity descriptors Inferential analyses were restricted to the same eight-member longitudinal panel and used Friedman tests with assessor as the blocking factor, followed by paired Wilcoxon signed-rank tests with Holm adjustment; Kendall's W was used as the omnibus effect size.
Microbiological analyses	Total viable count, yeasts, moulds/fungi, Staphylococcus, Enterobacteriaceae and aerobic sporulates. Reviewer-facing microbiological summaries use log10(CFU/g); values reported below the analytical threshold are retained as censored results rather than replaced by arbitrary numeric substitutions.
Chemical analyses	Protein, fat, saccharides, salt and related composition markers
Multivariate analyses	PCA1 for storage stability of the dry product; PCA2 for the rehydrated consumption-quality profile Primary PCA analyses used the balanced 3-18 month design at 5, 25 and 40 °C (108 formulation-by-month-by-temperature condition cells), with condition-level aggregation, z-standardization, ln-transformed η40 and no imputation.
**Experimental unit and replication**	Individually packaged pouches represented independent storage units for physicochemical and other applicable analyses. For physicochemical parameters summarized with n = 3, three independently stored pouches were analysed, with one analytical determination per pouch; repeated instrumental readings within a pouch (e.g., colour readings) were technical replicates. For rheology, two independently stored pouches from the same formulation × storage time × temperature condition were combined to form one composite sample; ≥3 rheological runs were performed on that composite and treated as technical replicates. These technical runs were averaged to one condition-level rheological value and were not treated as additional independent observations. Each formulation was prepared as one large production batch and then divided into the individual storage pouches.

**Sensory panel consistency**	The same eight assessors constituted the longitudinal panel used for inferential analysis. Additional students participated in selected sessions but did not evaluate the complete sample series and were therefore excluded from the repeated-measures inferential dataset.

Table S3. Overview of processed datasets retained by the authors.

The processed datasets summarized below are retained by the authors and are available from the corresponding author upon reasonable request; they are not submitted as a separate supplementary workbook.

Dataset / record	Content
S1_Physchem_Long	Processed long-format physicochemical dataset (aw, pH, moisture, colour parameters). Product and formulation labels are translated to English.
S2_Color_Lab_Long	Detailed CIELAB and ΔE*ab values for lyophilized and rehydrated products. Rehydrated ΔE values used in the revised PCA were reconciled against the validated colour source; the recovered values matched the existing clean records within rounding and were not imputed.
S3_Sensory_Means	Full 10-parameter sensory means by product, storage month, temperature and formulation. The final inferential sensory reanalysis was restricted to the consistent eight-assessor longitudinal panel; repeated-measures outputs are documented in the audited statistical reanalysis records retained by the authors.
S4_Rheology_Group	Group-level rheological summary across matrix, formulation, storage month and temperature. For integrated sensory-rheology modelling, rheological replicates were aggregated to formulation-by-month-by-temperature condition cells before modelling. Each condition-level rheological value is the mean of technical runs from one composite prepared from two stored pouches.
S5_Rheology_18M	Rheological endpoint table after 18 months. Endpoint n values denote technical runs of the two-pouch composite.
S6_Microbiology_OK	Final cleaned microbiological mean table for oat porridges. Reviewer reanalysis retains below-threshold values as censored log10 results and avoids unreported substitution when quantified and censored replicates are mixed.
S7_Microbiology_Rice	Final cleaned microbiological mean table for rice porridges. Reviewer reanalysis retains below-threshold values as censored log10 results and avoids unreported substitution when quantified and censored replicates are mixed.
S8_Chemistry	Chemical composition table extracted from the manuscript.
S9_Endpoint_18M_40C	Integrated endpoint quality profile after 18 months at 40 C.

Note	The processed physicochemical dataset uses moisture values recalculated from the original gravimetric records, with summary standard deviations recalculated consistently as sample SD (n - 1), and the corrected RK20 1M water-activity value (aw = 0.0846 ± 0.0011 (n = 3)).

Supplementary statistical and sensitivity notes

Because the same assessors evaluated all three formulations within each sensory session, formulation effects were re-analysed using Friedman tests with assessor as the blocking factor. Significant omnibus tests were followed by paired Wilcoxon signed-rank comparisons with Holm adjustment, and Kendall's W was used as the omnibus effect size. Across 560 session-specific Friedman tests, 224 had raw $p < 0.05$ and 154 pairwise formulation contrasts remained significant after Holm adjustment. As a broader multiplicity sensitivity analysis, Benjamini-Hochberg false-discovery-rate control applied across all 560 omnibus tests retained 116 significant tests; the most robust signals were concentrated in appearance, consistency, taste/aroma and overall evaluation.

Rheological temperature-ramp measurements were performed at a constant shear rate of approximately 77.3 s^{-1}. This condition was selected to approximate practical spoon mixing/stirring of rehydrated porridge while providing a standardized basis for comparison across formulations and storage conditions; it was not treated as an exact in-mouth shear rate.

For the sensory-rheology integration, ≥3 technical runs from each two-pouch rheological composite were first averaged to one formulation-by-storage-month-by-storage-temperature condition cell. Matrix-specific models in the balanced 3-18 month design (54 cells per matrix) related condition-level sensory scores to ln(η40) while adjusting for formulation, storage month and storage temperature; HC3 robust standard errors were used. Excluding the incomplete 9-month sensory sessions left all six primary ln(η40) coefficients non-significant, so the substantive conclusion was unchanged.

Supplementary physicochemical stability

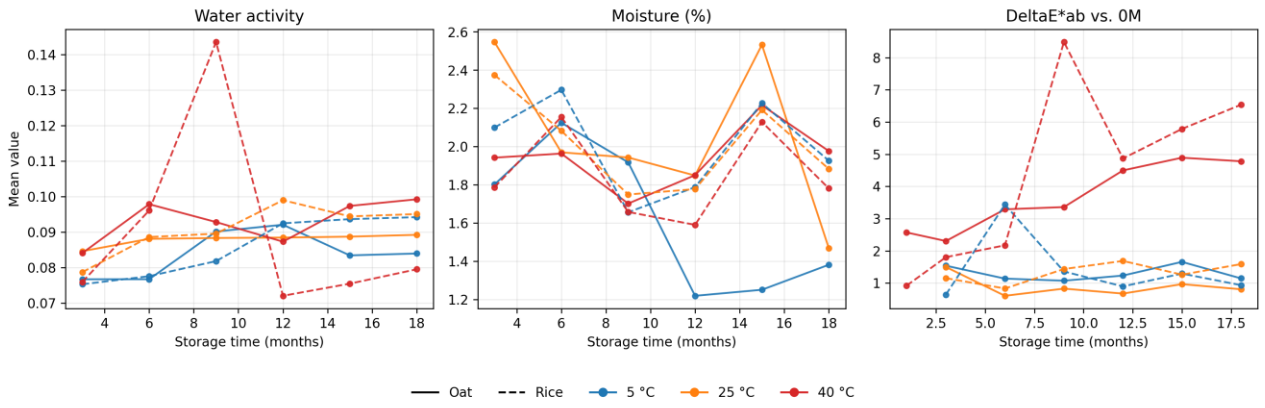

Figure S1. Trends of selected physicochemical stability markers during storage. Values are averaged across formulations within each matrix and storage temperature. Lines distinguish oat and rice matrices; colours distinguish storage temperatures.

Supplementary rheological detail

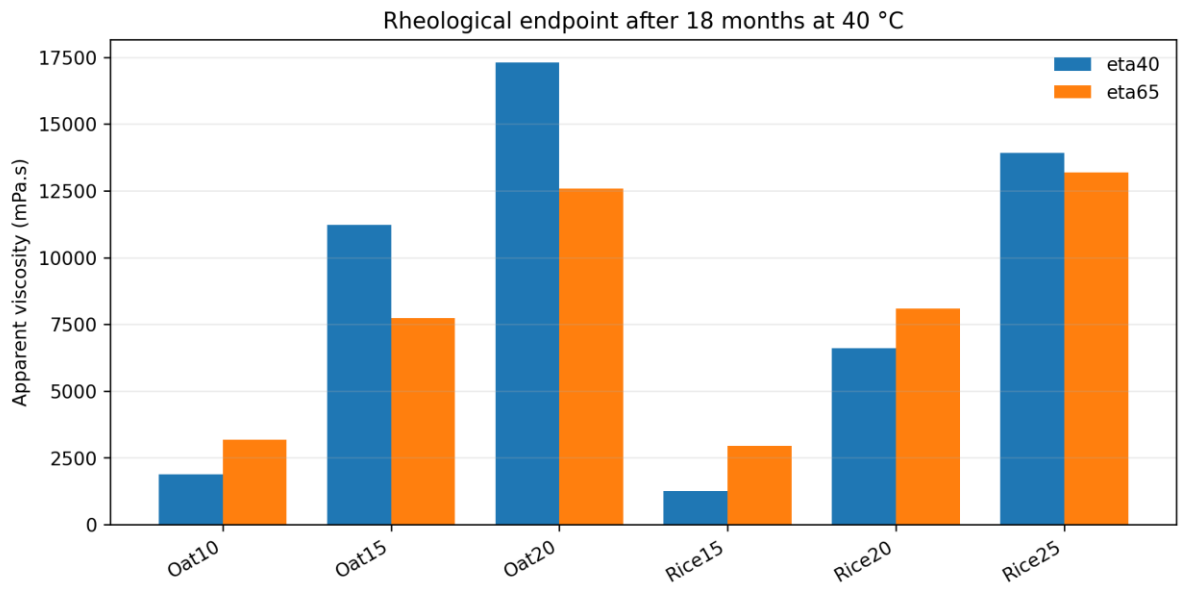

Figure S2. Apparent viscosity endpoint after 18 months of storage at 40 °C. Bars show apparent viscosity at 40 °C (η40) and 65 °C (η65) for each formulation. Each formulation shown here represents one composite prepared from two independently stored pouches; the displayed viscosity values are means of the technical rheological runs from that composite.

Supplementary chemical overview

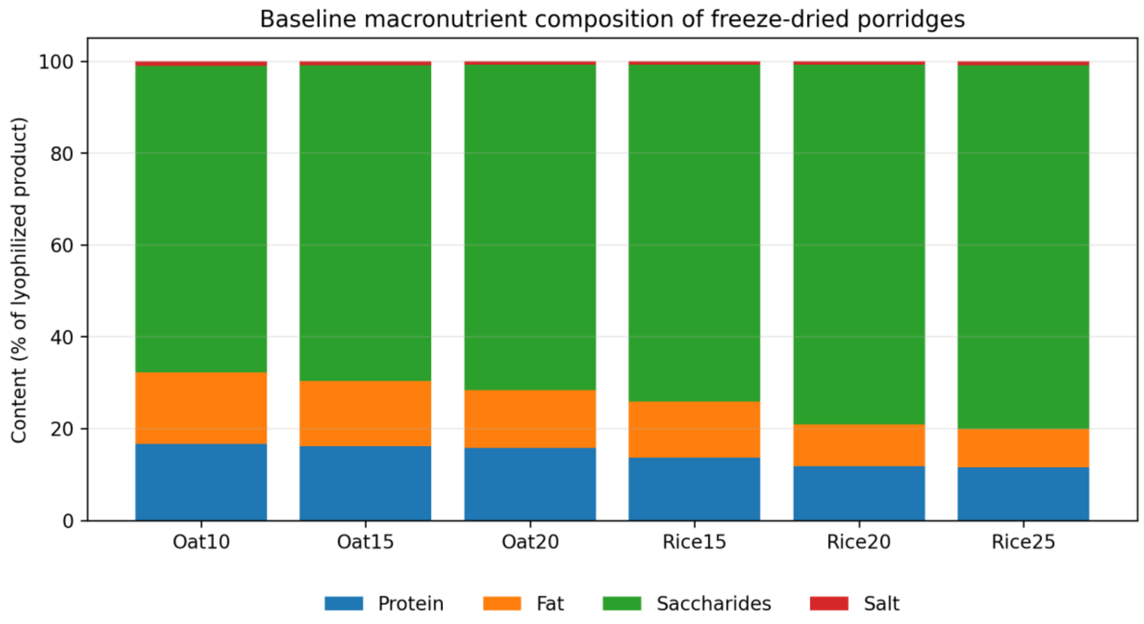

Figure S3. Baseline macronutrient composition of freeze-dried porridge formulations at 0M.

Supplementary PCA outputs

The revised PCA used the final QC-checked balanced condition-level dataset (n = 108; 3, 6, 9, 12, 15 and 18 months × 5, 25 and 40 °C × six formulations). Variables were z-standardized, η40 was natural-log transformed, and no missing value was imputed. PCA1 used water activity, pH, moisture, dry-product ΔE, ln(η40), TVR and VRI η40. PCA2 used pH, rehydrated ΔE, ln(η40), TVR, VRI η40 and the four principal positive sensory attributes. The rehydrated ΔE records were reconciled from the validated colour source rather than statistically imputed.

Table S4. PCA1 correlation loadings for the first two principal components (revised 108-condition analysis).

**Revised values from the audited 108-condition PCA1**		
**Variable**	**PC1 correlation loading**	**PC2 correlation loading**
Water activity (aw)	0.3197	0.5778
pH	-0.6472	0.1470
Moisture content	-0.4055	-0.2661
ΔE*ab of lyophilized product vs 0M	-0.0959	0.7825
Natural-log apparent viscosity at 40 °C [ln(η40)]	0.8960	-0.0654
Thermal viscosity response ratio (TVR)	-0.8216	0.2423
Viscosity retention index at 40 °C (VRI η40)	0.2040	0.6631

Table S5. PCA2 correlation loadings for the first two principal components (revised 108-condition analysis with rehydrated ΔE restored).

**Revised values from the audited 108-condition PCA2**		
**Variable**	**PC1 correlation loading**	**PC2 correlation loading**
pH	0.4441	0.5301
Natural-log apparent viscosity at 40 °C [ln(η40)]	-0.5872	-0.6953
Thermal viscosity response ratio (TVR)	0.3858	0.7645
Viscosity retention index at 40 °C (VRI η40)	0.0259	-0.1695
ΔE*ab of rehydrated product vs 0M	-0.2653	0.2750
Sensory appearance	0.9351	-0.1971
Sensory texture/consistency	0.9498	-0.1689
Sensory taste and aroma	0.8727	-0.3046
Sensory overall impression	0.8876	-0.2825

Table S6. PCA1 explained variance for the first five components (revised 108-condition analysis).

**Revised explained variance from the audited 108-condition PCA1**			
**Component**	**Eigenvalue**	**Explained variance (%)**	**Cumulative variance (%)**
PC1	2.2142	31.6310	31.6310
PC2	1.5412	22.0178	53.6488
PC3	1.0006	14.2939	67.9428
PC4	0.7819	11.1702	79.1129
PC5	0.7166	10.2371	89.3500

Table S7. PCA2 explained variance for the first five components (revised 108-condition analysis with rehydrated ΔE restored).

**Revised explained variance from the audited 108-condition PCA2**			
**Component**	**Eigenvalue**	**Explained variance (%)**	**Cumulative variance (%)**
PC1	4.0878	45.4200	45.4200
PC2	1.6932	18.8137	64.2337
PC3	1.1989	13.3213	77.5550
PC4	0.8348	9.2753	86.8304
PC5	0.6966	7.7404	94.5708

PCA sensitivity analysis. Excluding the incomplete 9-month sensory sessions produced loading-vector correlations of $r = 0.988$ (PC1) and $r = 0.748$ (PC2) for PCA1, indicating that the first component was highly stable whereas the second component should be interpreted more cautiously. PCA2 was highly robust to exclusion of 9 months, with loading-vector correlations of $r = 0.999$ for PC1 and $r = 0.998$ for PC2. Rehydrated ΔE was represented most strongly on PC3 (correlation loading approximately 0.695), supporting interpretation of colour drift as a partially distinct dimension from the principal sensory-rheology axis.
